# NK Cell-Mediated Processing Of *Chlamydia psittaci* Drives Potent Anti-Bacterial Th1 Immunity

**DOI:** 10.1038/s41598-019-41264-4

**Published:** 2019-03-18

**Authors:** Nadine Radomski, Kati Franzke, Svea Matthiesen, Axel Karger, Michael R. Knittler

**Affiliations:** 1 Institute of Immunology, Friedrich-Loeffler-Institut, Federal Research Institute of Animal Health, D-17493 Greifswald, Isle of Riems Germany; 2 Institute of Infectology, Friedrich-Loeffler-Institut, Federal Research Institute of Animal Health, D-17493 Greifswald, Isle of Riems Germany; 3 Institute of Molecular Virology and Cell Biology, Friedrich-Loeffler-Institut, Federal Research Institute of Animal Health, D-17493 Greifswald, Isle of Riems Germany

## Abstract

Natural killer (NK) cells are innate immune cells critically involved in the early immune response against various pathogens including chlamydia. Here, we demonstrate that chlamydia-infected NK cells prevent the intracellular establishment and growth of the bacteria. Upon infection, they display functional maturation characterized by enhanced IFN-γ secretion, CD146 induction, PKCϴ activation, and granule secretion. Eventually, chlamydia are released in a non-infectious, highly immunogenic form driving a potent Th1 immune response. Further, anti-chlamydial antibodies generated during immunization neutralize the infection of epithelial cells. The release of chlamydia from NK cells requires PKCϴ function and active degranulation, while granule-associated granzyme B drives the loss of chlamydial infectivity. Cellular infection and bacterial release can be undergone repeatedly and do not affect NK cell function. Strikingly, NK cells passing through such an infection cycle significantly improve their cytotoxicity. Thus, NK cells not only protect themselves against productive chlamydial infections but also actively trigger potent anti-bacterial responses.

## Introduction

NK cells play an important role in the immune response against various pathogens including chlamydia^1^. Through their interactions with other immune cells, they are important mediators between innate and adaptive immunity^2^. NK cells express a set of activating/inhibiting receptors^3^, which generate signals whose balance determines which cellular program is chosen^4^. They are activated by various cytokines^5^ resulting in the activation of phospholipase C (PLC). PLC generates two messengers, 1,2-diacylglycerol (DAG) and inositol 1,4,5-trisphosphate (IP3), which activate protein kinases C (PKCs) and mobilize Ca^2+^ from intracellular stores. DAG promotes PKCϴ translocation to membranes and phospho-activation, regulating NK-mediated effector functions^6^.

To detect and lyse target cells, NK cells use distinct mechanisms: Antibody-dependent cell-mediated cytotoxicity (ADCC) and natural cytotoxic activity^7^. In ADCC, the Fc part of target cell-bound IgG is recognized by the FcγRΙΙΙ receptor (CD16) on NK cells, upon which cytotoxic proteins are released in addition to IFN-γ. This leads to the cytotoxic killing of target cells^8^. No prior sensitization is needed for natural cytotoxicity, allowing for rapid detection/killing by this mechanism^8^. After direct contact with the target cell, secretory granules (containing granzymes and perforin) are released into the immunological gap^8^. Moreover, NK cells can kill via TNF family ligands^9^ as well as via the secretion of cytokines and chemokines^10^.

DAG-mediated activation of PKCs is sufficient to induce degranulation of NK cells, leading to the release of granzyme B^11^. Granzyme B is initially synthesized as an inactive precursor whose propeptide is removed by cathepsin C^12^, generating the enzymatically active protease. Perforin mediates the entry of activated granzyme B into the cytoplasm of target cells, where a large number of substrates are cleaved and apoptosis is induced^13^. Active granzyme B has also bactericidal activity^14,15^, processes cytokines^16^, and degrades extracellular matrix proteins^17^.

Upon establishing a chlamydial infection, the innate immune system provides an important stage in the defence against the bacteria. Epithelial cells, which are the initial targets for infection, are capable to trigger this early immune response^18^. Thus, it is well-known that *Chlamydia (C.) trachomatis*-, *C. muridarum*- and *C. psittaci*-infection in epithelial cells induce the production of pro-inflammatory cytokines^19,20^. In addition, secretion of IL-8 recruits innate immune cells such as dendritic cells (DCs) and NK cells^21^. During this immediate step, NK cells are among the first cells at the chlamydial infection site, as early as ≤24 hours post infection (hpi), and play an important role in the initial control of the immune response^22^. They are stimulated to secrete IFN-γ by infected DCs and epithelial cells, which produce IL-12 and IL-18, respectively^23^. NK cells have been shown to be the source of early *in vivo* IFN-γ production^1^ and display functional activation when PBMCs (peripheral blood mononuclear cells) are stimulated with *Chlamydia* (*C.) trachomatis*^24^. Moreover, infection of epithelial cells with *C. trachomatis* makes them susceptible to NK cell lysis^24^. NK cells seem to be critically involved in the defence against *C. trachomatis* genital tract infections, as their depletion leads to an exacerbated course of infection with a diminished cellular immune response^1^. They may also play an important role in the defence against chlamydial lung infections, as NK cell-depleted mice show more severe disease following *C. muridarum* lung infection with decreased Th17 and Th1 cells correlated with reduced IL-12, IL-17, IL-22, and IFN-γ^25^. IFN-γ restricts chlamydial growth by different mechanisms, e.g. by increasing phagocytic activity of macrophages^26^. Furthermore, IFN-γ down-regulates the transferrin receptor preventing the iron transport into the cell, which might be required for chlamydial survival^27^. Further, IFN-γ-mediated induction of indoleamine 2,3-dioxygenase (IDO) depletes cellular tryptophan that is essential for chlamydia (e.g. *C. psittaci*)^28^. Moreover, IFN-γ activates the production of reactive oxygen and nitrogen species that kill *C. trachomatis* in neutrophils and macrophages^29^. Finally, NK cell-secreted IFN-γ not only is important in inhibiting the growth of chlamydia but also directs DCs to mount an adaptive Th1 immune response^22^.

Previously, we had demonstrated that *C. psittaci*-infected DCs harbour cell-autonomous self-protection mechanisms that disintegrate chlamydial inclusions and routes them for xenophagic degradation, leading to the generation of MHC I presented antigens^30^. Since both DCs and NK cells serve as essential part of a crucial “first line of defence” and are among the initial immune cells encountered by chlamydia during infection^1^, we asked whether NK cells are also capable of being infected by the bacteria and whether they mount self-defence strategies against such infections. Unravelling the interaction between NK cells and chlamydia will help to better understand the initial defence strategies and molecular key mechanisms during the innate immune response against bacterial pathogens.

## Results

### NK cells prevent the intracellular establishment and growth of chlamydia

**Figure 1:**
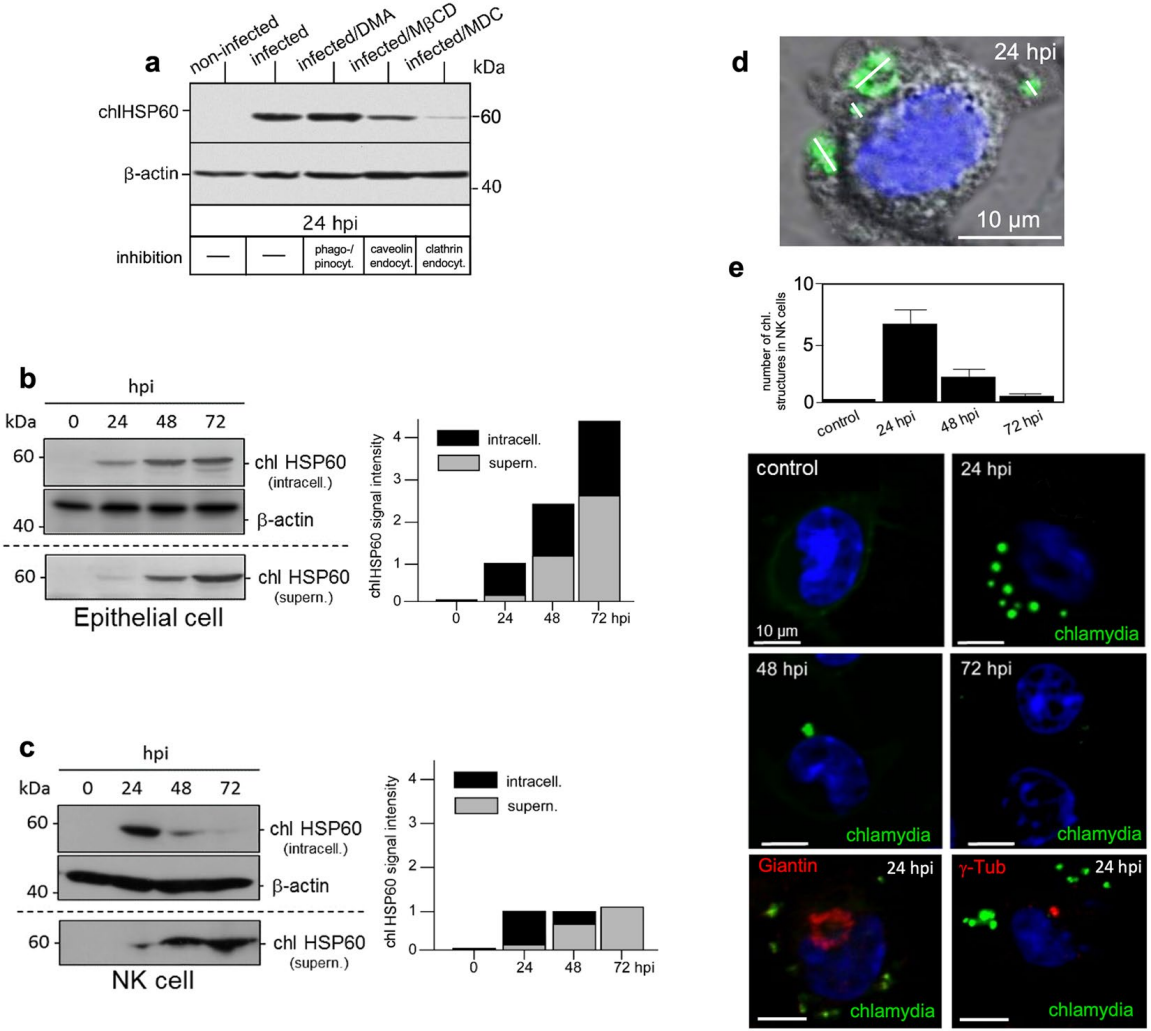
Chlamydial infection of KY-2 cells. (**a**) KY-2 cells were infected for 24 h in the presence of inhibitors blocking cellular uptake (100 µM DMA, 10 mM MβCD, 200 µM MDC)^94^. Lysates were analysed by Western blot probed for chlHSP60 and β-actin. (**b**,**c**) Western blots (left) of infected KY-2 and MN-R cells showing chlHSP60 in lysates (intracell.) and culture supernatants (supern.). β-actin served as a loading control. chlHSP60 signals were determined by densitometric analysis (right). chlHSP60 signal intensities in cells (black column part) and supernatants (grey column part) are shown. The signal of total chlHSP60 at 24 hpi was set to 1. (**d**) Depicts an infected KY-2 cell (24 hpi) stained for chlamydial LPS (green) overlaid on the phase-contrast image. DNA is visualized with DAPI (blue). The size of bacterial structures are indicated by white lines. (**e**) KY-2 cells were infected or not with chlamydia (0–72 hpi, MOI 40) and stained for chlamydia (green) and DNA (DAPI, blue). A total of 200 cells was counted from 10 random fields of view (63x objective) to determine the number of chlamydial structures in infected NK cells (0–72 hpi). The results are shown in the top panel of (**e**). Infected KY-2 cells (MOI 40 and 24 hpi) were also co-stained for chlamydia (green), DNA (DAPI, blue), Giantin (Golgi, red) or γ-tubulin (MTOC, red). 55–75% of the KY-2 cells were infected. (**a,b,c**) Depict cropped blots obtained by each protein evaluation. Full-length blots are shown in the Supplementary Figs S4, S5 and S6, respectively.

**Figure 2:**
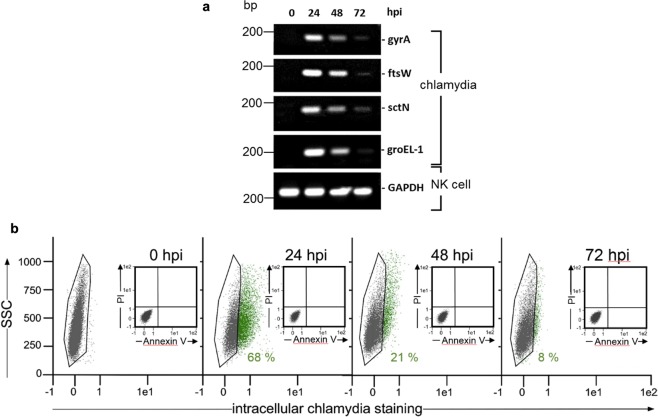
Chlamydial load of infected KY-2 cells. (**a**) RT-PCR of chlamydial factors. PCR amplification products were separated on a 1% agarose gel (amplicons of mRNA of gyrA, ftsW, sctN, groEL-1 of chlamydia-infected KY-2 cells (MOI 40)). GAPDH served as control. (**b**) Flow cytometric analysis of infected KY-2 cells (0–72 hpi). To detect/quantify chlamydia-positive NK cells (green), the negative cell population (black) was identified and gated via corresponding non-infected controls and then subtracted from the total cell population. Flow cytometric analysis of necrotic/apoptotic KY-2 cells during infection was performed by using annexin V-FITC kit from Miltenyi Biotec and propidium iodide (see inserts in Fig. 1b). The original agarose gel image (**a**) is shown in the Supplementary Fig. S7.

By using KY-2 cells (established murine NK cell line with homogeneous/consistent culture properties)^31^ and the non-avian *C. psittaci* strain DC15^32^ as a suitable model system for chlamydial infection, we first investigated whether and by what cellular uptake mechanism KY-2 cells are infected with chlamydia. Therefore, the cells were incubated with chlamydia (MOI 40) for 24 h in the presence of inhibitors blocking different cellular uptake mechanisms (see methods). Lysates of infected and non-infected cells were analysed by Western blot probed for chlamydial (chl)HSP60 as a proxy for bacterial growth^30^ (Fig. 1a). The uptake of chlamydia was strongly affected by monodansylcadaverine (MDC) indicating that, like in epithelial cells^33^, clathrin-mediated endocytosis is critically involved in the chlamydial engulfment. Macropinocytosis/phagocytosis and caveolae-dependent endocytosis seemed negligible for the infection. Next, we compared chlamydial infections of epithelial and NK cells. MN-R (immortalized epithelial cells from newborn mice, see methods) and KY-2 cells were incubated with chlamydia (0–72 h) and infection was monitored via Western blotting using chlHSP60 (Fig. 1b,c). In infected epithelial cells (MOI 20), the expression of chlHSP60 correlated directly with the course of infection (24–72 h). In cell extracts and corresponding culture supernatants, we found a characteristic time-dependent increase of the chaperone (Fig. 1b, left panel). Thus, bacteria grow well in these host cells and are continuously released into the culture medium (Fig. 1b, right panel). In KY-2 cells (MOI 40), we also observed a marked increase of chlHSP60 in cell extracts at ≥24 hpi. This suggests that KY-2 cells are indeed infected by chlamydia (Fig. 1c, left panel). In contrast to epithelial cells, intracellular chlHSP60 decreased at ≥48 hpi and was barely detectable at 72 hpi (Fig. 1c, right panel). This is unlikely to reflect the degradative elimination of the pathogen, because the intracellular decrease of chlHSP60 correlated tightly with a time-dependent increase of the chaperone in the medium (Fig. 1c, right panel). Thus, the total chlHSP60 levels (intracellular plus supernatant) remained nearly constant for KY-2 cells at ≥24 hpi, whereas total chlHSP60 levels progressively increased for epithelial cells (Fig. 1b,c, right panels). We next analysed infection of KY-2 cells using immunofluorescence microscopy (Fig. 1d,e), RT-PCR (Fig. 2a) as well as flow cytometry (Fig. 2b). In agreement with Fig. 1c, infected KY-2 cells established no perinuclear inclusions (with characteristic Golgi/MTOC association) during the course of infection (Fig. 1d,e). In contrast to infected epithelial cells^30^, multiple peripheral small bacteria-positive vacuoles with diameters of 1–3 μm were observed (Fig. 1d,e). Between 24–48 hpi only a few and at 72 hpi nearly no chlamydial structures were found within KY-2 cells (Fig. 1e). The time-dependent disappearance of bacteria in infected NK cells was also confirmed by RT-PCR (Fig. 2a) monitoring various chlamydial transcripts (gyrA, ftsW, sctN, and groEL-1, see methods). Infected KY-2 cells displayed a very low number of necrotic/apoptotic cells at all time points, showing that these cells are not dying and thereby disappearing from the culture (Fig. 2b, insets). Correlating with the results obtained by Western blotting (Fig. 1c), we observed a maximum of chlamydia-positive cells at ≥24 hpi followed by a continuous reduction of intracellular bacteria between 48–72 hpi. KY-2 cells allow infection but not growth of internalized chlamydia. This suggests that NK cells get rid of the bacteria within 72 hpi by releasing them into the extracellular environment.

### Chlamydia-infected NK cells display functional maturation

**Figure 3:**
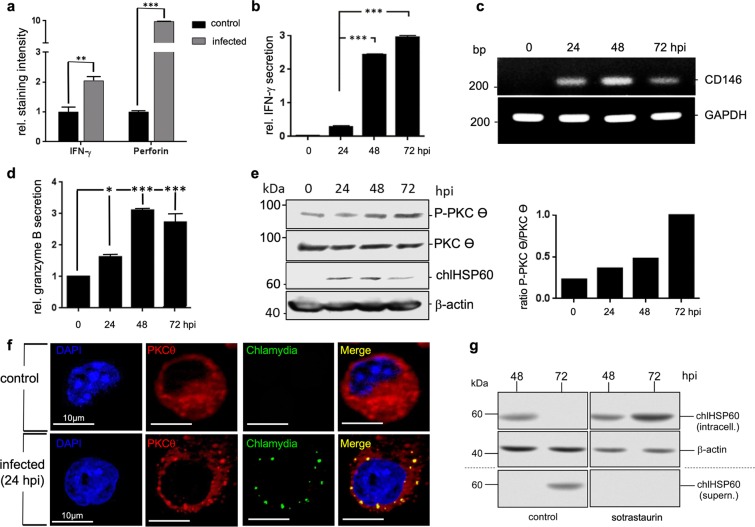
Functional activation of KY-2 cells during chlamydial infection. (**a**) Flow cytometric analysis of IFN-γ and perforin expression of infected KY-2 cells (MOI 40, 48 hpi). The plot shows the staining intensity of IFN-γ- and perforin-positive KY-2 cells compared with the value for non-infected cells, which was set to 1. Statistical analysis was performed as described in methods (**p < 0.01 and ***p < 0.001 vs. control (non-infected), n = 3). (**b**) ELISA of IFN-γ secretion of infected KY-2 cells. The plot displays the relative amount of IFN-γ secretion as means ± SD. The maximum value at 72 hpi was set to 3. (**c**) RT-PCR of CD146 transcript levels in uninfected and infected KY-2 cells (0–72 hpi). Amplicons were separated on a 1% agarose gel. GAPDH served as a control. The plot in (**d**) shows the relative granzyme B secretion from infected KY-2 cells (MOI 40, 0–72 hpi) measured by ELISA. The values obtained for non-infected cells were set to 1 (*p < 0.05 and ***p < 0.001 vs. control (non-infected), n = 3). (**e**) Western blot of PKCΘ phospho-activation during KY-2 cell infection (left panel). KY-2 cells were infected or not with chlamydia (MOI 40) for 0–72 h and analysed by Western blots probed for P-PKCϴ, PKCϴ, and chlHSP60. β-actin served as a loading control. After densitometric analysis, the P-PKCϴ/PKCϴ ratio was plotted for the different time points of infection (right panel). (**f**) Immunofluorescence showing the co-localization between PKCϴ (red) and chlamydia (green) in infected NK cells (MOI 40, 48 hpi). (**g**) Western blot of chlHSP60 in infected KY-2 cells (MOI 40) and culture supernatants in the presence of sotrastaurin (250 nM). β-actin served as a loading control. (**e and g**) Depict cropped blots obtained by each protein evaluation. Full-length blots and the original agarose gel image (**c**) are shown in the Supplementary Figs S8, S9, and S10, respectively.

Next, we were interested to test whether transient chlamydial infection is also capable of activating NK cells. KY-2 cells were infected with chlamydia for 48 h and expression of perforin and IFN-γ were measured by flow cytometry (Fig. 3a). In parallel, we analysed IFN-γ secretion by ELISA (Fig. 3b) and expression of the NK cell maturation marker CD146^34^ by RT-PCR (Fig. 3c). All assays demonstrated that infected KY-2 cells are activated, functionally mature, and secrete IFN-γ (Fig. 3a–c). Moreover, infected KY-2 cells actively degranulated, shown by the release of granzyme B (Fig. 3d). Accordingly, PKCϴ underwent phospho-activation (Fig. 3e), a step thought to trigger downstream signalling, activation, and degranulation in NK cells^6^. PKCϴ displayed significant co-localization with chlamydial structures (Fig. 3f). The PKCϴ inhibitor sotrastaurin^35^ strongly suppressed chlamydial release, causing bacterial accumulation within the KY-2 cells (Fig. 3g).

### Infected NK cells release chlamydial structures by degranulation

**Figure 4:**
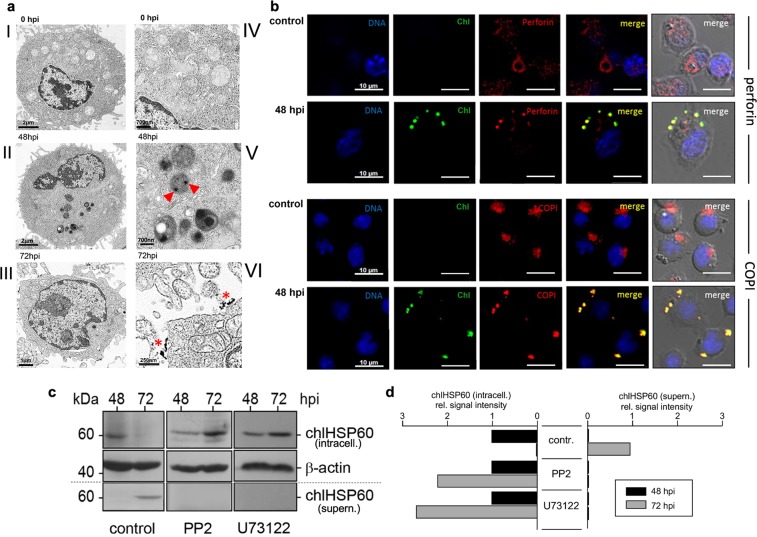
Chlamydial structures and their intracellular colocalization with secretory granules in infected KY-2 cells. (**a**) TEM of non-infected (0 hpi, I and IV) and infected (48 and 72 hpi, MOI 40) KY-2 cells (II, III, V and VI). Arrowheads and asterisks indicate chlamydial/granular remnants inside vacuolar structures and on the cell surface (72 hpi). (**b**) Immunofluorescence of perforin (red, upper panels) or COPI (red, lower panels) and chlamydia (green) in infected (MOI 40, 48 hpi) and non-infected cells. DNA (blue) was stained with DAPI. Cross-reactivity of anti-perforin and anti-α-COP with chlamydia was checked with isolated/purified bacteria (Supplementary Fig. S11). (**c**) Western blot of chlHSP60 expression in infected KY-2 cells (MOI 40) in the presence of PP2 (0.1 μM) or U73122 (10 μM). Cell lysates were analysed by Western blots probed for chlHSP60 and β-actin. (**d**) After densitometric analysis, chlHSP60 signals obtained for infected cells (48 hpi) were set to 1 (left: intracellular; right: supernatant). (**c)** Depicts cropped blots obtained by each protein evaluation. Full-length blots are shown in the Supplementary Fig. S12.

Degranulation of NK cells is a process of regulated secretion^36^ of granules with a size between 0.2–1 μm^37^. This would allow the uptake of chlamydial elementary bodies (EBs, Ø 0.2–0.3 μm) and reticular bodies (RBs, Ø 0.5–1 μm) into these vacuoles and their release by degranulation. In view of a nearly synchronous release of chlamydia (Fig. 1c) and granzyme B (Fig. 3d), we asked whether chlamydial structures are localized within secretory granules of NK cells. KY-2 cells were infected with chlamydia (MOI 40) and analysed by electron microscopy. In non-infected KY-2 cells, secretory granules were detectable as type II vacuolar structures^38^ containing lamellar and vesicular material (Fig. 4a-I/-IV). In contrast, in infected cells (48 hpi), most of the secretory granules were filled with electron-dense material (Fig. 4a-II) and large round particles (Ø 0.2–0.3 µm) corresponding in size to chlamydial EBs. A further granule population contained electron dense material of smaller size (Ø 50–90 nm) suggesting that some of the engulfed pathogens had been structurally disintegrated (Fig. 4a-V, red arrowheads). All these granules disappeared at 72 hpi and electron dense material with an amorphous structure appeared on the cell surface (Fig. 4a-III/-VI, red asterisks). This suggests that bacterial material is in physical contact with secretory granules and that chlamydial release is linked to the degranulation in infected KY-2 cells. Consistent with this, chlamydial structures co-localized with perforin-positive granules, whereas perforin resided in circular structures near the nucleus in non-infected cells (Fig. 4b, top panels).

The coat protein complex I (COPI) seems to promote *C. trachomatis* entry into the host cell downstream of cell surface attachment^39^. A critical role of COPI in chlamydial infection is also shown by the identification of α, β, β′, γ and ξ-COP as important host factors in *C. caviae* infection^40^. Moreover, it has been suggested that COPI-positive vesicles might deliver nutrients to chlamydial inclusions^41,42^. COPI envelope proteins have also been found within the membranes of secretory granules^43^ and seem to play an important role in regulated secretion^44^. COPI proteins in non-infected KY-2 cells were in close proximity to the nucleus (Fig. 4b, bottom panels) associated with the Golgi^45^, whereas in infected KY-2 cells COPI proteins were found within chlamydial structures (Fig. 4b, bottom panels). This suggests that COPI-positive inclusions are actively fusing with secretory granules. To investigate whether chlamydia are released via degranulation, KY-2 cells were infected with chlamydia and treated with PP2 and U73122 blocking PLC/DAG-dependent degranulation^46,47^. Indeed, both inhibitors suppressed chlamydial release (Fig. 4c, middle/right panel and Fig. 4d).

**Figure 5:**
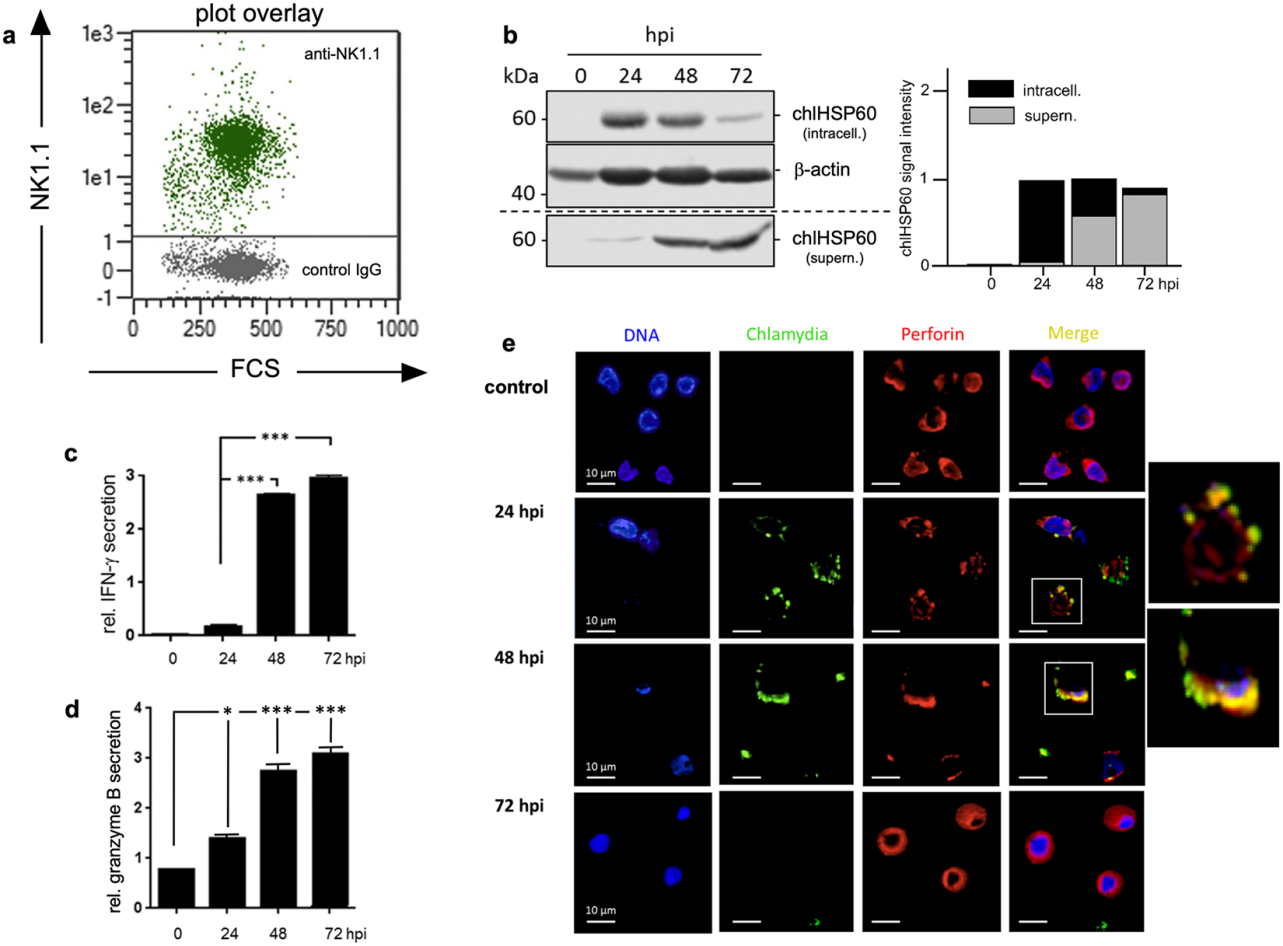
Chlamydial infection of primary NK cells. (**a**) MACS-isolated primary NK cells examined via flow cytometry to determine the proportion of NK1.1-positive cells isolated from the spleen of C57BL/6 mice (dot plot overlay of IgG isotype control (grey) and NK1.1-specific surface staining (green)). (**b**) Western blot of infected primary NK cells (MOI 40) probed for chlHSP60 in cell lysates (intracell.) and culture supernatants (supern.) (left). After 3 h of infection, extracellular chlamydia were removed by washing with PBS (0 hpi). Cells were further cultivated in fresh medium and lysed at various time points. Corresponding culture supernatants were centrifuged and obtained pellets were also collected. β-actin served as a loading control. chlHSP60 signal intensities (intracell. and supern.) were determined by densitometric analysis (right). The graph shows chlHSP60 in cells (black column part) and supernatants (grey column part). Total chlHSP60 at 24 hpi was set to 1. (**c**) ELISA of IFN-γ release by infected primary NK cells (MOI 40) (left panel). The plot displays the relative amount of IFN-γ secretion as means ± SD. The maximum value at 72 hpi was set to 3. (**d**) The plot shows the relative granzyme B secretion in infected primary NK cells (MOI 40, 0–72 hpi) measured by ELISA. The values for non-infected controls were set to 1. Statistical analysis was performed as described in methods (*p < 0.05 and ***p < 0.001 vs. control (non-infected), n = 3). (**e**) Immunofluorescence demonstrating the co-localization between perforin (red) and chlamydia (green) in infected primary NK cells (MOI 40, 24–72 hpi). (**b**) Depicts cropped blots obtained by each protein evaluation. Full-length blots are shown in the Supplementary Fig. S13.

KY-2 is a well established model system for NK cell function^31^. However, we were also interested to see whether primary NK cells show the same properties when infected with chlamydia. Primary NK cells were isolated from the spleens of C57BL/6 mice with ≥96% purity (Fig. 5a). Western blots of chlHSP60-stained cell extracts and culture supernatants from infected primary NK cells demonstrated the same efficient chlamydial release into the environment (Fig. 5b). This suggests that immortalized and primary NK cells employ the same defence strategy (uptake and subsequent release) during infection. In support of this, infected primary NK cells also displayed enhanced IFN-γ secretion and granzyme B degranulation (Fig. 5c,d). Moreover, immunofluorescence studies revealed that infected primary NK cells also displayed strong co-localization between bacteria and perforin-positive granules (Fig. 5e, 24 and 48 hpi). As in KY-2 cells, small chlamydial structures were located close to the plasma membrane, did not form perinuclear inclusions, and disappeared completely at 72 hpi (Fig. 5e).

### Bacterial infection/release can be undergone repeatedly and do not affect NK cell function

**Figure 6:**
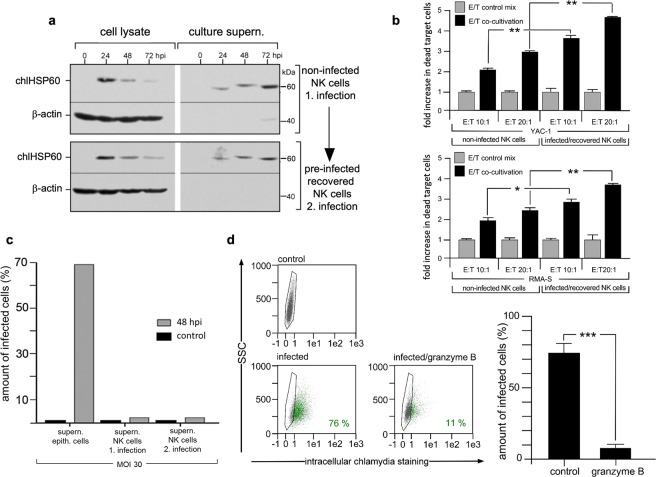
Pre-infection/recovery/reinfection and its impact on KY-2 cells and chlamydial infectivity. (**a**) For Western blot, KY-2 cells were pre-infected (MOI 40) for 72 h, washed, recovered for 72 h and then reinfected. At different time points, cells were lysed and pellet fractions of corresponding culture supernatants were collected. β-actin served as a loading control. Primary infection of KY-2 cells is shown in the upper part of (**a**). (**b**) Flow cytometry of target cell killing by pre-infected/recovered and/or non-infected KY-2 cells. Adherent KY-2 cells were co-cultured for 4 h with YAC-1 or RMA-S cells with an effector/target ratio (E:T) of 10:1 and 20:1, respectively. The suspension target cells were carefully separated from KY-2 cells by aspiration and stained with PI. For controls, fixed KY-2 cells were mixed with target cells immediately before staining. The graph shows the fold increase of permeabilized target cells after co-cultivation with KY-2 cells compared to the control cell mix (mean values from three measurements ± SD, *p < 0.05 and **p < 0.01 vs. control (non-infected), n = 3). (**c**) Flow cytometry of the infectivity of culture supernatants (supern.) from infected epithelial and KY-2 cells. KY-2 cells (non-infected and recovered after primary pre-infection) and MN-R cells were infected (MOI 30) or not for 48 h. Culture supernatants were used for incubation with BGM reporter cells. The graph shows the relative amount of chlamydia-positive cells (48 hpi). (**d**) Flow cytometry of epithelial cell infection after treatment of EBs with granzyme B. EBs were incubated for 4 h at 37 °C with proteolytically activated granzyme B or left untreated. EBs were washed and used for the infection of MN-R cells (48 hpi, MOI 30). The result is depicted as a histogram plot (right panel) (***p < 0.001 vs. control (non-infected), n = 3). (**a**) depicts cropped blots obtained by each protein evaluation. Full-length blots are shown in the Supplementary Fig. S14.

NK cells seem to clear their chlamydial infection via degranulation. Therefore, we asked whether NK cells are able to repeat this after recovery from a previous infection. Additionally, we asked whether this strategy has an effect on the cytotoxicity of NK cells. KY-2 cells were first infected with chlamydia for 72 h (74% cell infection at 24 hpi), washed and cultivated in fresh medium for a further 72 h. Next, these cells were re-infected for 0–72 h (75% cell infection at 24 hpi) and analysed in Western blots probed for chlHSP60. As seen in Fig. 6a (lower part), infection of pre-infected/recovered KY-2 cells resulted in a time-dependent reduction of chlHSP60 in the cell extracts from 24–72 hpi. This reduction was paralleled by an increase of chlHSP60 in the corresponding culture supernatants. When comparing the results of the first infection (Fig. 6a upper part) with those of the subsequent second infection (Fig. 6a, lower part), the kinetics of chlamydial release were almost identical. We also performed killing assays with non- and pre-infected/recovered KY-2 cells (Fig. 6b). As target cells for killing assays we used the suspension cell lines YAC-1 (upper part) and RMA-S (lower part)^48,49^. After 4 h of co-cultivation of target and effector cells, dead target cells were identified via flow cytometry using propidium iodide (PI) staining^50^. As a control, target cells were mixed with adherent paraformaldehyde fixed KY-2 cells shortly before analysis (control mix) (Fig. 6b). For non-infected KY-2 cells, the amount of killed target cells increased depending on the respective effector/target ratios (10:1 and 20:1) by factors of 2–3 when compared to the corresponding control mixes (Fig. 6b). In the killing assays with pre-infected/recovered KY-2 cells, cytotoxicity was enhanced (3–4.5 fold when compared to the control mixes) suggesting that infection and recovery of NK cells have a positive effect on cytotoxicity.

### Granzyme B causes inactivation of NK cell-released chlamydia

Figure 4a suggests that chlamydia may lose their structural integrity during their “transient visit” in NK cells. Thus, we also analysed the infectivity of NK cell-released chlamydia. Reporter cells were incubated (48 h) with culture supernatants of infected KY-2 cells (1. or 2. infection, see Fig. 6a) or infected epithelial cells (control) and analysed by flow cytometry. NK cell-released chlamydia did not infect the reporter cells (Fig. 6c). Next, we investigated whether granule-localized proteases are responsible for the loss of bacterial infectivity. Enriched EBs were treated with recombinant active granzyme B and then used for epithelial cell infection (MOI 30) monitored by flow cytometry (Fig. 6d, left panels). As seen in Fig. 6d (right panel), granzyme B-treatment of EBs resulted in a dramatic reduction of infectivity. Moreover, we observed that infected KY-2 cells treated with cell-permeable granzyme B inhibitors release chlamydia with detectable infectivity (Supplementary Fig. S1). Taken together, this indicates that granzyme B is involved in the degradative inactivation of chlamydia before the organisms are released via degranulation.

### NK cell-released chlamydia induce production of pathogen-specific Th1-related antibodies

**Figure 7:**
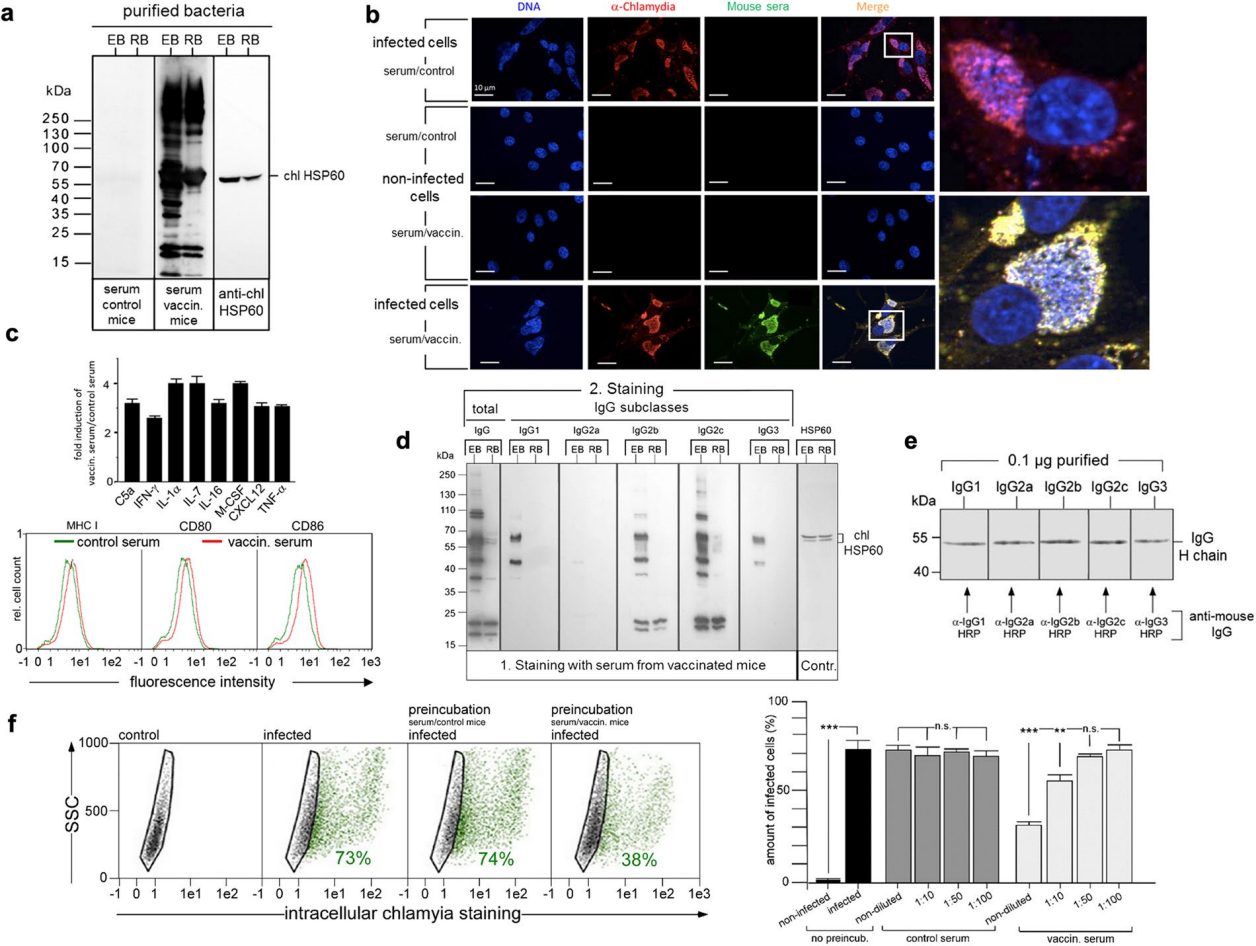
Characterization of the antibody response triggered by KY-2 cell-released chlamydia. (**a**) C57BL/6 mice were immunized with non-infectious chlamydia from KY-2 cells (controls were treated with PBS). Vaccinated and control sera were analysed in Western blots (**a**) and immunofluorescence studies (**b**). Enriched EBs/RBs were used for Western blots (**a**) The immunostaining (**a**,**b**) was performed with control and/or vaccinated serum. Anti-mouse pan-IgG-HRP (**a**) or -FITC (**b**) were used as secondary antibodies. Western blots were also probed for chlHSP60. (**b**) BGM reporter cells were infected or not (MOI 20, 48 hpi) and stained with anti-chlamydial antibody (red), control or vaccinated serum (green). (**c**) Expression of cytokines/chemokines after immunization with KY-2 cell-released chlamydia (upper panel). Sera from vaccinated and control mice were analysed via cytokine array (R&D Systems). The plot shows the group of cytokines/chemokines with altered levels (mean ± SD, n = 3 independent experiments). For the DC maturation assay (lower panel), primary mouse BMDCs were cultured for 48 h in medium containing 30% serum from vaccinated or control mice and analyzed by flow cytometry. (**d**) Enriched EBs and RBs were analysed in Western blots, which were first incubated with vaccinated serum and then probed with secondary anti-mouse IgG-HRP antibodies specific for different IgGs. chlHSP60 staining served as a control. (**e**) Comparability of IgG subclass recognition was checked with purified IgGs. (**f**) Flow cytometry of BGM reporter cells infected with EBs (48 hpi, MOI 20), which were pretreated (4 h) or not with vaccinated and control serum (non-diluted or diluted). The plot (bottom, left panel) displays the amount of chlamydia-positive cells as means ± SD (n.s.: not significant; **p < 0.01 and ***p < 0.001) vs. control (non-infected), n = 3). (**a,d,e**) Depict cropped blots obtained by each protein evaluation. Full-length blots are shown in the Supplementary Figs S15, S16 and S17, respectively.

We next analysed the ability of NK cell-released non-infectious chlamydia to induce an anti-chlamydial immune response. C57BL/6 mice (n = 3) were immunized with three consecutive doses of purified non-infectious chlamydia (in sterile PBS) released from KY-2 cells (72 hpi). Control mice (n = 3) were treated in parallel with sterile PBS. The sera of the mice (control and vaccinated) were tested for the presence of chlamydia-specific IgGs. Extracts of enriched EBs and RBs were analysed by Western blots stained with control and/or vaccinated mouse sera (Fig. 7a). Blots were additionally probed for chlHSP60 (Fig. 7a, right panel), which confirmed that comparable quantities of EBs and RBs were loaded. For the control serum no or only weak signals could be detected (Fig. 7a, one representative example out of three tested sera), whereas the vaccinated serum specifically stained a broad range of distinct bacterial polypeptides. Although far more proteins were recognized for enriched EBs than for enriched RBs, some of the signals were also visible for both (Fig. 7a, one representative example out of three tested sera). Further immunofluorescence studies revealed that the serum from vaccinated animals binds to chlamydia-specific structures in the context of infected cells (Fig. 7b, one representative example shown out of three tested sera). Thus, NK cell-released non-infectious chlamydia have immunogenic properties and induce production of pathogen-specific antibodies.

Next, we studied which kind of immune response is triggered by NK cell-released non-infectious chlamydia during immunization. Therefore, we analysed the cytokine/chemokine profile following the vaccination procedure by using a mouse array assay (R&D Systems). A clear trend toward an increase of Th1-related/driving cytokines (IFN-γ, TNF-α, IL-7, IL-1α, IL-16), chemokines (CXCL12) and factors (C5a) was seen for the group of induced immunological mediators (Fig. 7c, upper panel). Moreover, consistent with Th1-polarisation, primary murine DCs that were cultivated with serum from vaccinated mice showed a detectable upregulation of surface expressed MHC I, CD80 and CD86 (factor 2–3) when compared to DCs cultured with serum from control mice (Fig. 7c, lower panel). Based on this, we focused on the detection of different IgG subclasses, which are known to have distinct immunological properties. Four IgG subclasses exist in mice, of which IgG1 indicates a Th2 response (humoral immunity), while IgG2a/c, b, and IgG3 are characteristic for Th1 responses (cellular immunity)^51^. In C57BL/6 mice, IgG2c functionally replaces IgG2a, which is not present due to genomic deletion^52^. To identify the different IgGs involved in the recognition of chlamydia, lysate extracts of EBs and RBs were analysed in Western blots probed with sera from vaccinated and control mice (Fig. 7d, one representative example shown out of three sera). Secondary staining was performed with five different IgG subclass-specific antibodies. Comparability of IgG subclass recognition was confirmed with purified IgGs in a parallel Western blot (Fig. 7e). Staining with anti-chlHSP60 and anti-pan IgG was used as an additional control for equal sample loading^53^ and EB/RB discrimination, respectively (Fig. 7d leftmost/rightmost panel). Analysis of the different IgGs revealed a pronounced EB-staining for IgG2c followed by IgG2b (Fig. 7d). Again, a strong staining of EBs was observed, whereas RBs were recognized to a much weaker extent. IgG1 and IgG3 showed no (RBs) or detectably reduced staining (EBs) for chlamydial antigens (Fig. 7d). The IgG1 signal appears to be somewhat stronger than the IgG3 signal. Thus, the obtained staining intensities (IgG2c > IgG2b > IgG1 ≥ IgG3) suggest that Th2 immunity is not excluded from the reaction, but that the immune response is apparently dominated by IgG2b/IgG2c, reflecting a Th1-mediated immune response^51^.

Finally, we examined whether the vaccinated serum can neutralize chlamydial EBs, affecting cellular uptake and infection. Isolated/purified EBs were pre-incubated or not with control or vaccinated serum (2 h/4 °C, 2 h/37 °C). Binding of the IgG subclasses to the EBs was controlled by antibody binding/EB sedimentation assays (Supplementary Fig. S2). Pre-incubated EBs were then used for the infection of epithelial reporter cells (MOI 5). 48 hpi cellular infection was analysed by flow cytometry (Fig. 7f). Pretreatment of EBs with control serum (non-diluted, 1:10, 1:50 and 1:100 in PBS) had no detectable influence on the infection of epithelial cells (infection rates of ≥74%) (Fig. 7f, left and right panel). However, chlamydia, which were pretreated with non-diluted vaccinated serum, showed a significantly reduced infection of cells when compared to the controls (≥50% reduction) (Fig. 7f, left and right panel). In the case of the 1:10 dilution, a reduction of about 30% was observed. This suggests that non-infectious chlamydia released by infected NK cells induce a Th1 response with neutralizing IgGs, which reduce bacterial infection of target cells.

## Discussion

NK cells are a crucial part of the innate immune system and play an important role in the defence against microbial infections^7^. There are only few studies on NK cell infection, and these studies mostly focus on viral pathogens. For instance, Renoux and coworkers demonstrated uptake of human papillomavirus by NK cells, which led to increased cytotoxicity and cytokine production^54^. In contrast, influenza A virus negatively affected the function/viability of infected NK cells^55^. Here we show that *C. psittaci* is able to infect immortalized and primary NK cells and uncover a novel cellular self-defence mechanism. NK cells use their secretory granules to first inactivate the intruders and then release non-infectious, but immunogenic bacterial material via degranulation. The early steps of chlamydial “rerouting” in NK cells are likely related to the initial events in infected epithelial cells, characterized by the fusion of *C. trachomatis*-positive vacuoles with Golgi‐derived vesicles^56^. Via modifications of their inclusion membrane, *C. trachomatis* escape the endo-/lysosomal pathway^56^ and prevent their “recycling” back to the plasma membrane^57^. Eventually, chlamydial compartments migrate along microtubules to the central perinuclear region where homotypic fusion and formation of a large perinuclear inclusion occurs^58^. These later events of intracellular chlamydial development do not happen in infected NK cells (Figs 1–5). Instead, bacterial inclusions remain dispersed in multiple small vacuoles (Ø 1–3 μm) closely beneath the plasma membrane (Figs 1–5).

After entering NK cells, bacterial structures are COPI positive (Fig. 4). This is reminiscent of the situation in epithelial cells, in which COPI promotes *C. trachomatis* entry^39^ and COPI vesicles^40^ might deliver nutrients to chlamydial inclusions^41^. ADP-ribosylation factor 1 (Arf1), a key regulator of membrane organization, localizes to chlamydial inclusions (*C. trachomatis*, *C. muridarum* and *C. pneumoniae*)^59^ and regulates the recruitment of COPI vesicles^60^. COPI is crucial for regulated secretion processes^44^ and COPI and Arf1 are present in the membranes of secretory granules^43,61^. It is tempting to speculate that early chlamydial vacuoles after entering NK cells hijack the host machinery to recruit COPI vesicles and thereby “accidentally” fuse with COPI-positive secretory granules. These granules contain a battery of enzymes^8^ with anti-microbial properties^62^ that prevent enclosed chlamydia from growing and initiating transport towards the microtubule organizing centre (MTOC)/Golgi (Fig. 1). Indeed, for *Escherichia coli, Listeria monocytogenes*, and *Mycobacterium tuberculosis*, it has been demonstrated that granzyme B cleaves a highly conserved set of bacterial proteins. These proteins play important roles in the biosynthesis/metabolism of pathogens and are crucial for bacterial survival^15^. In accordance with this, chlamydia released from KY-2 cells as well as granzyme B-treated EBs (Fig. 6) display drastically reduced infectivity, while treatment of KY-2 cells with granzyme B inhibitors rescues chlamydial infectivity (Supplementary Fig. S1). This suggests that granzyme B might play a critical role in the inactivation of chlamydia inside secretory granules. Moreover, granules of NK cells contain cathepsins as well as various other lysosomal hydrolases and anti-microbial proteins^63,64^. These compartments have an acidic pH and combine the degradative function of conventional lysosomes with the capacity to undergo regulated exocytosis^8^. The function of secretory lysosomes in immune cells can be exocytic (towards target cells) - or internal, whereby they fuse with phago-/endosomal compartments that have engulfed pathogens^65^ ^933^. In macrophages, which use the internal pathway, chlamydial growth is efficiently suppressed via late endo-/lysosomal targeting^66^. Inside these phagocytes, chlamydia show phenotypic similarities to the bacterial structures observed in infected NK cells^66^. In both cell types chlamydia localize to dispersed acidic lysosomal compartments in which the pathogen is inactivated. Thus, macrophages and NK cells may use similar strategies to reroute internalized bacteria into the endo-/lysosomal pathway in which the bacteria are targeted to conventional or secretory lysosomes. In macrophages the recruitment of IFN-inducible guanylate binding proteins (GBPs) to early inclusions is involved in the rerouting of chlamydia for lysosomal degradation^67^. It will be interesting to see whether this is also true for chlamydia-infected NK cells.

NK cells are activated by a variety of bacterial pathogens. This process requires toll-like receptor (TLR)-mediated activation of antigen presenting cells (APCs)^68^. Since NK cells express TLRs and other pattern recognition receptors (PRRs)^69^, a direct activation of the cells is also possible. Indeed, there is evidence that TLR2-dependent recognition of *Mycobacterium bovis* can lead to direct activation of NK cells^69^. This is consistent with our own findings demonstrating that NK cells are directly activated by chlamydial infection (Fig. 3). Moreover, infected KY-2 cells display phospho-activation of PKCϴ, which is associated with chlamydial structures (Fig. 3). PKCϴ is critically involved in activation/degranulation processes of NK cells^70^. The kinase functions as a signalling intermediate downstream of NK cell-activating receptors through its well-documented role in the PLC pathway^71^. PLC activation leads to the formation of DAG and IP3, which as second messengers increase intracellular Ca^2+^ and activate PKCs. Activated NK cells from PKCϴ^−/−^ mice are defective in killing, indicating that activation/degranulation depends on this kinase^70,71^. Moreover, PKCϴ may be directly implicated in NK cell degranulation elicited by specific killer-activating receptors (KARs)^6^. The critical role of the PLC/PKC pathway in degranulation was originally demonstrated via phorbol-12-myristate-13-acetate (PMA)/ionomycin-induced stimulation^72^. These studies suggested that NK cell-mediated degranulation is directly triggered via PKCϴ activation. Our findings on the phosho-activation and inclusion-co-localization of PKCϴ in infected KY-2 cells are consistent with observations that PKCs are activated during chlamydial infection^73^ and that PKCs co-localize with chlamydial structures (Fig. 3)^74^. We hypothesize that infection-triggered PKCϴ-activation on inclusions initiates degranulation and chlamydial release. Accordingly, the PKCϴ-inhibitor sotrastaurin^35^ and the degranulation inhibitors PP2 and U73122 impair this process (Figs 3, 4).

IFN-γ production is a crucial step in the immune defence against various pathogens^75^. During infection, NK cells are the major source of early IFN-γ production^1^. IFN-γ inhibits chlamydial growth (e.g. *C. trachomatis*)^76^ and is crucial for clearing the infection *in vivo*^77^. Various experimental systems have shown that the absence of IFN-γ leads to reduced Th1-related IgG levels, spread of infection, and defects in protective immunity^23^. NK cell activation by *C. trachomatis*-primed APCs results in IFN-γ secretion and lysis of infected target cells^24^. Our findings suggest that the production of IFN-γ starts as soon as infection of NK cells occurs (Fig. 3). A clear benefit of this would be a fast downstream-activation of local immunity against chlamydia.

Our observation that pre-infected/recovered KY-2 cells retain their ability to release inactivated *C. psittaci* (Fig. 6) suggests that NK cells act as “reusable” effectors. NK cells are very long-lived cells capable of repeatedly participating in immune responses and generating immune memory^78^. Such “trained immunity” is observed upon antigen treatment as well as cytokine-mediated stimulation^78^ and has been described in the context of bacterial infections^79^. Supporting this, we found improved cytotoxicity of pre-infected/recovered KY-2 cells when compared to non-infected controls (Fig. 6). Repetitive activation via PKC-induction may eventually cause a hyporesponsive cell state and/or a situation of anergic unresponsiveness^80^. Indeed, the activity/function of NK cells isolated from *C. trachomatis*-infected patients is seemingly impaired^81^. Specifically, a drastic decrease in the killing activity was observed and reduced TNF-α/IFN-γ release after incubation with target cells. Clearly, further studies are required to understand the functional modulation of NK cells following multiple/repetitive chlamydial infections.

Immunization of C57BL/6 mice with chlamydial material previously released from KY-2 cells elicited an antibody response that was dominated by Th1-related IgG2b/IgG2c (Fig. 7) indicating a cellular type of immune reaction. DCs exposed to non-viable/non-infectious *C. trachomatis* express inflammatory and immunomodulatory molecules and these DCs confer resistance to chlamydial challenge after adoptive transfer^82^. In fact, DCs pulsed with dead *C. trachomatis* are characterized by increased MHC II expression and IL-12 secretion^83^, which is required for Th1 differentiation^84^. Moreover, inactivated *C. trachomatis* together with GM-CSF drive the accumulation of DCs at the site of administration in infected mice^85^. This correlates then with the development of protective immunity^85^, in which Th1 cell-secreted cytokines and the corresponding IgG class switch^86^ cause the dominance of IgG2b/IgG2c. Future experiments will explore whether NK cell-released chlamydia confer immune protection.

Different roles are proposed for antibodies in immunity to chlamydia. Neutralizing antibodies may play a crucial role by preventing the attachment of *C. trachomatis* to epithelial cells^87^. Indeed, our results show that anti-chlamydial antibodies induced by NK cell-processed bacteria neutralize infections *in vitro* (Fig. 7). More indirect antibody-based mechanisms (*i.e*. via Fc receptors) also seem to be important. ADCC for instance, is involved in the elimination of *C. trachomatis* infections^88^ and a role for ADCC in the clearance of chlamydia (*C. trachomatis* and *C. psittaci*) is supported by the detection of antigens on the surface of infected cells^89^. Antibody-mediated complement-dependent cytotoxicity (CDC) may also play a role in the elimination of chlamydia-infected cells. We note that the Th1-related IgGs, which we identified in our experiments (Fig. 7), are all known to be very efficient in mediating ADCC and/or CDC^44^.

**Figure 8:**
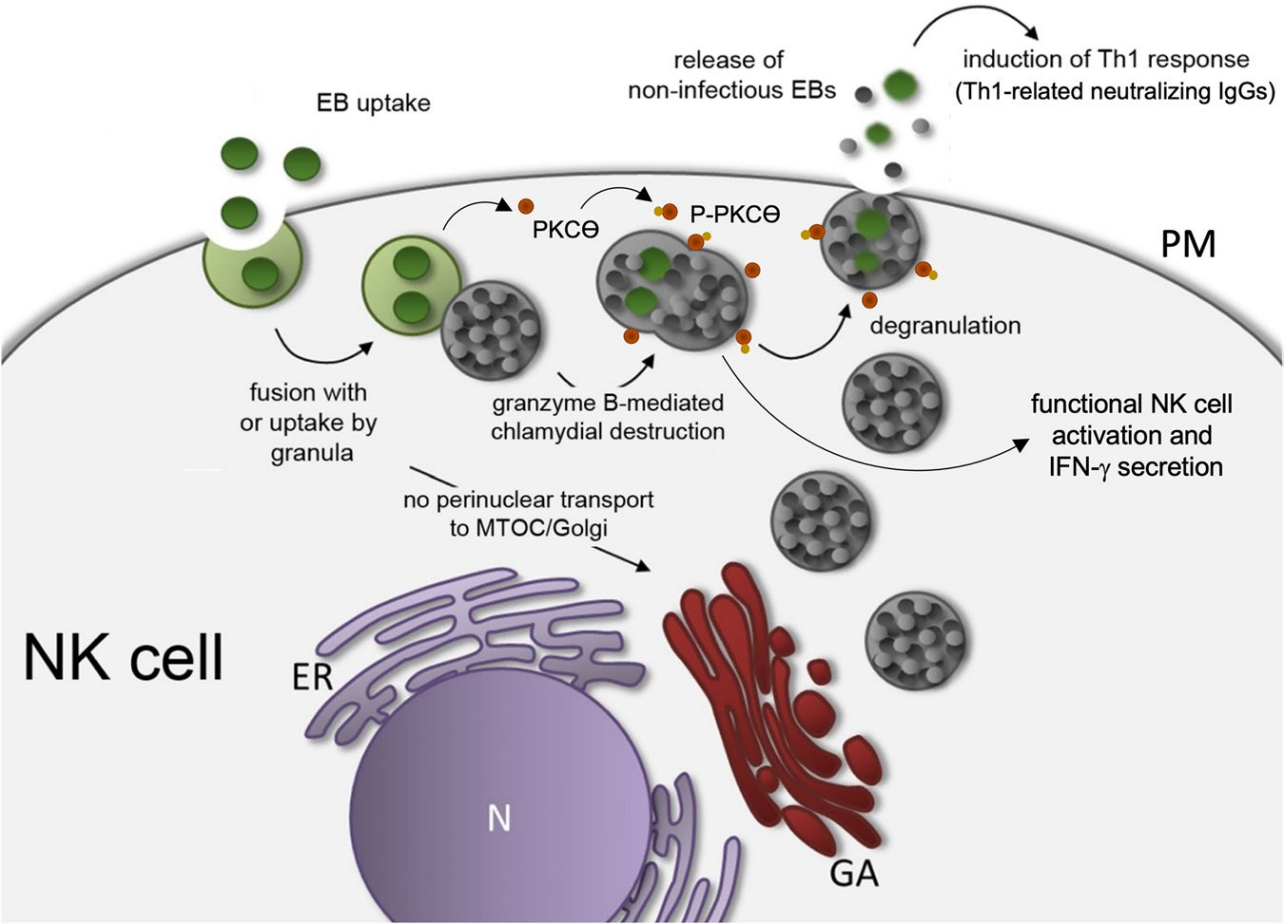
Postulated working model for the anti-chlamydial defence of NK cells and the immune response triggered by the released inactivated non-infectious chlamydia. The depicted model shows the transient chlamydial infection of NK cells triggering activation, cytokine secretion, bacterial granule fusion and chlamydial release (N, nucleus; GA, Golgi apparatus; ER, endoplasmic reticulum; PM, plasma membrane).

We postulate a model (Fig. 8), in which upon uptake into NK cells the developmental cycle of the chlamydia is stopped, no productive inclusions form and no transport to the MTOC/Golgi region occurs (Fig. 1). Instead, the infection activates NK cells via PKCϴ, driving increased IFN-γ secretion accompanied by enhanced perforin and CD146 induction. Chlamydial structures fuse with granules that are released via degranulation. It might be possible that chlamydial particles are already transformed from EBs to replicative/non-infectious RBs. However, our electronic microscopy findings (Fig. 4) suggest that such EB/RB transformation does apparently not occur in infected NK cells. We rather speculate that during this process, chlamydial proteins from EBs are degraded via granzyme B, thereby generating non-infectious, but immunogenic bacterial forms, which upon release induce a Th1-response associated with neutralizing IgGs. It is tempting to speculate that the pathway represents a general mechanism to not only eliminate intracellular bacteria, but to also mount potent anti-bacterial Th1 immunity. Our results give the first hint for the orientation of the involved immune response, but it is clear that further studies have to be performed to show whether and to what extent this newly discovered process plays a role for the immune defence in natural infections. NK cells may harbour significant immunotherapeutic potential in the treatment of bacterial infections, which we believe should be explored. Unravelling the precise mechanisms driving NK cell-triggered immunity may help to build new innovative platforms for the development/production of novel cell-based vaccines that use infected NK cell cultures instead of pathogen-treated epithelial cell systems.

## Methods

### Cell culture

The murine NK cell clone KY-2^31^ (a kind gift from W. Yokoyama, Washington University School of Medicine) was cultivated at 37 °C and 7.5% CO_2_ in RPMI1640 medium supplemented with 2 mM L-Glutamine, 10% FCS, β-mercaptoethanol (10 µM), 200 U/ml IL-2). Depending on growth rate and cell density, the cells were passaged every 3–5 days. Immortalized epithelial cells from newborn mice (MN-R cells) were obtained from the Collection of Cell Lines in Veterinary Medicine (CCLV) of the Friedrich-Loeffler-Institut (CCLV-RIE #282).The epithelial African green monkey kidney cell line BGM was obtained from the National Reference Laboratory for Chlamydiosis of the Friedrich-Loeffler-Institut, Jena (CCLV-RIE #136). Cells were grown at 37 °C/7.5% CO_2_ in IMDM cell culture with 5% FCS. YAC-1 (ATCC TIB-16), an NK-sensitive murine lymphoma cell line was obtained from American Type Culture Collection. In addition, we also used the NK-sensitive mouse RMA-S T lymphoma cell line (TAP2-deficient and antigen presentation-defective) for our NK cell assays (a kind gift from J.C. Howard, Institute for Genetics - University of Cologne, Germany & Instituto Gulbenkian de Ciência, Portugal). Primary BMDCs (C57BL/6 mice, 8-week-old) were produced after 7–14 days from bone marrow cells cultured in GM-CSF (5–10 ng/ml)-containing medium (IMDM) as described by Winzler *et al*.^90^ and assessed for purity by flow cytometry (CD11c, CD40, CD86, CD80 and MHC I/II staining). Different inhibitors were used to block cellular uptake mechanisms (DMA, dimethylamiloride: inhibition of macropinocytosis/phagocytosis; MβCD, methyl-β-cyclodextrin: inhibition of caveolae-dependent endocytosis; MDC, monodansylcadaverine: inhibition of clathrin-dependent endocytosis). 1-[6-[((17β)-3-Methoxyestra-1,3,5[10]-trien-17-yl)amino]hexyl]-1H-pyrrole-2,5-dione (U73122, 10 μM) and pyrazolopyrimidine (PP2, 0.1 μM) were used to block PLC/DAG-dependent cellular degranulation.

### Antibodies

Antibodies against chlamydial LPS, P-PKCϴ, PKCϴ, Giantin (Golgi marker), γ-tubulin (MTOC marker), NK1.1 (NK cell marker), IFN-γ, perforin (secretory granule marker) and α-COP were obtained from Abcam and CellSignaling. Anti-chlamydial HSP60 and LPS were purchased from Acris and Santa Cruz Biotechnology. Secondary (anti-mouse IgG and anti-mouse IgG1, 2a, 2b, 2c and 3) and isotype-control antibodies were purchased from Dianova, Thermo Fisher, and BioLegend.

### NK cell isolation from spleens of uninfected mice

After isolation of the spleens from uninfected female C57BL/6 mice (8-week-old), these were first transferred to 15 ml tubes and stored on ice. To release the cells, the spleens were cut into sections and passed through a cell sieve (70 μm) with slight pressure. The cell suspension was centrifuged (10 min, 300 × g, 4 °C.) and washed with PBS. Subsequently, NK cells were isolated using the mouse NK cell isolation kit from Miltenyi Biotec. All “non-NK cells” were bound by a mix of biotin-coupled antibodies and removed with anti-biotin antibodies from the cell suspension. Separation was carried out by applying a magnetic field through the MACS (Magnetic Activated Cell Sorter) from Miltenyi Biotec. Eluted NK cells were collected in culture medium containing IL-2 (200 U/ml). To determine the purity of the isolated NK cells, the cells were stained with a PE-coupled anti-NK1.1 antibody and measured in the MACSQuant flow cytometer (Miltenyi Biotec). This final control revealed that 96% of the purified cells were positive for NK1.1.

### Western blotting

Cells were lysed on ice in RIPA buffer (150 mM NaCl, 50 mM Tris-HCl, 1% NP-40, 0.25% Na-deoxycholate, and complete protease inhibitor (Roche), 50 mM NaF) with 4 M urea. After centrifugation (14.000 rpm, 30 min, 4 °C), postnuclear supernatants were analysed in Western blots as described before^30^. The used SDS PAGE protein markers were from Serva and ThemoFisher Scientific. Fluorographs were scanned and quantified with GelEval 1.32 (FrogDance Software).

### Chlamydia

The non-avian *C. psittaci* strain DC15^32^ was grown in BGM cells with chlamydial EBs and RBs purified by discontinuous density-gradient ultracentrifugation^91^ using Visipaque (Nycomed). Briefly, BGM cells were cultivated in antibiotic-free medium and confluent cultures were infected with 5 × 10^7^ inclusion forming units (IFUs). After 48 h cultivation at 37 °C and 7.5%, CO_2_ chlamydia-containing cells were harvested and the bacterial suspension was sonicated three times for 10 sec at 100 watts in an ultrasonic bath. After centrifugation (4.000 × g, 3 min, 4 °C) the supernatant was carefully transferred to ultracentrifuge tubes. Then, the suspension was underlaid with Visipaque solutions of different concentrations (2 ml 8% solution, 3 ml 15% solution followed by 5 ml 30% solution). Afterward, the tubes were centrifuged at 40.000 × g for 50 min and 4 °C. The pellet fraction was resuspended in PBS and used for a second ultracentrifugation whereby the obtained fraction was again carefully underlaid with different Visipaque solutions (1 ml 8%, 1 ml 15%, 1 ml 30%, 12 ml 36%, 8 ml 40%, and 5 ml 47%). After the second ultracentrifugation (50.000 × g, 50 min, 4 °C), enriched EBs^92^ were found between the 40% and 47% layer, while enriched RBs^92^ were located in the 36% layer. Fractions containing EBs and RBs were diluted in PBS and centrifuged again (30.000 × g, 50 min, 4 °C). Finally, pellets of enriched EBs and RBs were resuspended in sucrose-phosphate-glutamic acid buffer (SPGA) and stored at −70 °C. The purification/enrichment of EBs and RBs in the two fractions was visualized and checked by TEM. The results are depicted in Supplementary Fig. S3.

IFUs were determined by immunostaining (IMAGEN kit, Oxoid). Unless indicated otherwise, cells were infected with EBs at an MOI of 20–40. The percentage of infected cells (KY-2, primary NK cells, MN-R and BGM) in culture was determined by flow cytometry.

### Flow cytometry and colorimetry

Flow cytometry was performed as described previously^30^. Cells were analysed on a MACSQuant analyzer (Miltenyi Biotec). Viability was assessed with trypan blue. For chlamydial staining and titer determination, cells were fixed with 2% paraformaldehyde, permeabilized in PBS/0.5% saponin/0.5% BSA at RT for 30 min and immunostained with the IMAGEN kit (Oxoid).

### Immunofluorescence microscopy

For fluorescence microscopy, cells were grown on coverslips and fixed for 20 min in 2% paraformaldehyde, quenched with 3% BSA, permeabilized with 0.1% saponin (Sigma-Aldrich), and incubated serially with the indicated primary and corresponding secondary antibodies. Images were taken with an Axiovert 200 M/ApoTome microscope and a confocal Exciter laser scanning microscope (Zeiss)^30^. Colocalization was measured using AxioVision colocalization and Zen 2009 software (Zeiss). Pearson coefficients were calculated using the CoLocalizer Express software (CoLocalization Research Software).

### IFN-γ and granzyme B ELISA

To detect IFN-γ quantitatively in the cell culture supernatant of chlamydia-infected or uninfected NK cells, the IFN-γ Platinum ELISA from eBioscience was used. For the analysis, 1 × 10^5^ primary and immortalized NK cells were cultured in 12-well plates and infected with chlamydia for 24–72 h. Cell culture supernatants (50 µl undiluted supernatant as well as 1:100 and 1:500 in PBS) were analysed by ELISA according to the manufacturer’s instruction. For the quantitative detection of granzyme B in culture supernatants of chlamydia-infected NK cells, the mouse granzyme B Platinum ELISA from eBioscience was used. Therefore, 5 × 10^5^ immortalized or primary NK cells were cultured and infected or not with chlamydia for 24–72 h. At the respective time points, cell culture supernatants were collected, centrifuged at RT for 5 min at 1.300 rpm and then analysed via ELISA according to the manufacturer’s instructions. The colorimetric reactions were measured at a wavelength of 450 nm on a Sunrise Remote ELISA Reader (Tecan).

### RT-PCR

Total RNA from cells was isolated and analysed by semi-quantitative RT-PCR. The respective PCR primer pairs were: 5′‐GCGAAGCATCGTAAATGTGC‐3′, 5′‐AGCCGAA GTTTCCTTGACCAT‐3′ (*C. psittaci* gyrA, DNA replication); 5′‐TTGTTCCCTGCGT CGCTATC‐3′, 5′‐AAAAGCTATTACGGCTGCGGA‐3′ (*C. psittaci* ftsW, cell division); 5′‐CAACAGGTAGCAGAATCCGGA‐3′, 5′‐CTCTTCGCTGATAAGTTGGCCA‐3′ (groEL-1, chaperone/protein folding); 5′‐TGGTCTGGGAGAACCTATCG‐3′, 5′‐TCTGGCGACTGT GAGCATAC‐3′ (*C. psittaci* sctN, T3SS); 5′-TCACAGTCAGTCCTCACACCAG-3′, 5′-CAGATCGATGTATTTCTCTCCATCTC-3′ (mouse CD146, NK cell activation) and 5′-CACCTTCGATGCCGGGGCTG-3′, 5′-TGTTGGGGGCCGAGTTGGGA-3′ (mouse GAPDH, loading control). The MassRuler DNA ladder from ThermoFisher Scientific was used as a molecular weight marker in agarose gels. For semi-quantification of the PCR-amplificates, digital images of agarose gels were densitometrically analysed by using the software ImageStudioLite 4.0.2.1 (LI-COR Biosciences).

### Immunization of C57BL/6 mice with non-infectious Chlamydia from NK cells

8-week-old C57BL/6 mice were immunized with non-infectious chlamydia isolated from 1 × 10^6^ NK cells. For this purpose, the mice were immunized with a bacteria/PBS suspension of 200 μl intraperitoneally (i.p.), followed by two further immunizations on day 14 and 28. In the control mice (littermates), the three immunization steps were carried out in parallel with sterile PBS only. Seven days after the final immunization, the mice were sacrificed for complete blood collection. In the non-immunized animals, the spleens were additionally prepared. All animal procedures were approved by the local District Government (State Office for Agriculture, Food Safety, and Fishery in Mecklenburg-Western Pomerania - LALFF M-V) and were carried out according to the guidelines of the German law for the protection of animal life (LALLF M-V registration number: 7221.3-2-042/17, FLI No.: FLI 28/17).

### Transmission electron microscopy

For the TEM analysis, 5 × 10^5^ NK cells were infected with *C. psittaci* (MOI 40). After different time points (0, 48, and 72 hpi), cells were treated with fixing solution (2.5% glutaraldehyde buffered in 0.1 M sodium cacodylate (pH 7.2), 300 mosmol, Merck), carefully removed from the bottom of the culture flasks with a cell scraper, centrifuged (300 × g, 5 min, 4 °C) and embedded in low-melting-point agarose (Biozym). Small pieces were postfixed in 1.0% aqueous OsO_4_ and stained with uranyl acetate. After stepwise dehydration in ethanol, the cells were cleared in propylene oxide, embedded in Glycid Ether 100 (Serva), and polymerized at 60 °C for 3 days^93^. Ultrathin sections counterstained with uranyl acetate and lead salts were analysed with a Tecnai-Spirit TEM (FEI).

### Statistical analysis

Analysis of the obtained data is shown as the mean ± SD of three individual experiments and was estimated using GraphPad Prism 6 (GraphPad Software). Data were analysed by t-test and one-way analysis of variance (ANOVA) followed with Dunnett’s and/or Tukey’s post hoc test (n.s.: not significant; *p < 0.05; **p < 0.01 and ***p < 0.001).

## Supplementary information

NK Cell-Mediated Processing Of Chlamydia psittaci Drives Potent Anti-Bacterial Th1 Immunity

## Data Availability

All data generated or analysed during this study are included in this published article (and its Supplementary Information files).
