# Supplementary material for: NK Cell-Mediated Processing Of Chlamydia psittaci Drives Potent Anti-Bacterial Th1 Immunity
Source: Sci Rep. 2019 Mar 18;9:4799. doi: 10.1038/s41598-019-41264-4 (PMC6423132; doi:10.1038/s41598-019-41264-4)
Supplement: Supplementary file 1 — NK Cell-Mediated Processing Of Chlamydia psittaci Drives Potent Anti-Bacterial Th1 Immunity [file 41598_2019_41264_MOESM1_ESM.pdf]

Supplementary Information

NK Cell-Mediated Processing Of *Chlamydia psittaci* Drives Potent Anti-Bacterial Th1 Immunity

Nadine Radomski[1], Kati Franzke[2], Svea Matthiesen[1], Axel Karger[3], and Michael R. Knittler[1]*

[1]Institute of Immunology, Friedrich-Loeffler-Institut, Federal Research Institute of Animal Health,
D-17493 Greifswald - Isle of Riems, Germany

[2]Institute of Infectology, Friedrich-Loeffler-Institut, Federal Research Institute of Animal Health,
D-17493 Greifswald - Isle of Riems, Germany

[3]Institute of Molecular Virology and Cell Biology, Friedrich-Loeffler-Institut, Federal Research Institute of Animal Health,
D-17493 Greifswald - Isle of Riems, Germany

***Corresponding author:**
Dr. Michael R. Knittler
Friedrich-Loeffler-Institut
Institute of Immunology
Federal Research Institute of Animal Health
D-17493 Greifswald - Isle of Riems, Germany
michael.knittler@fli.de

Fig. S1

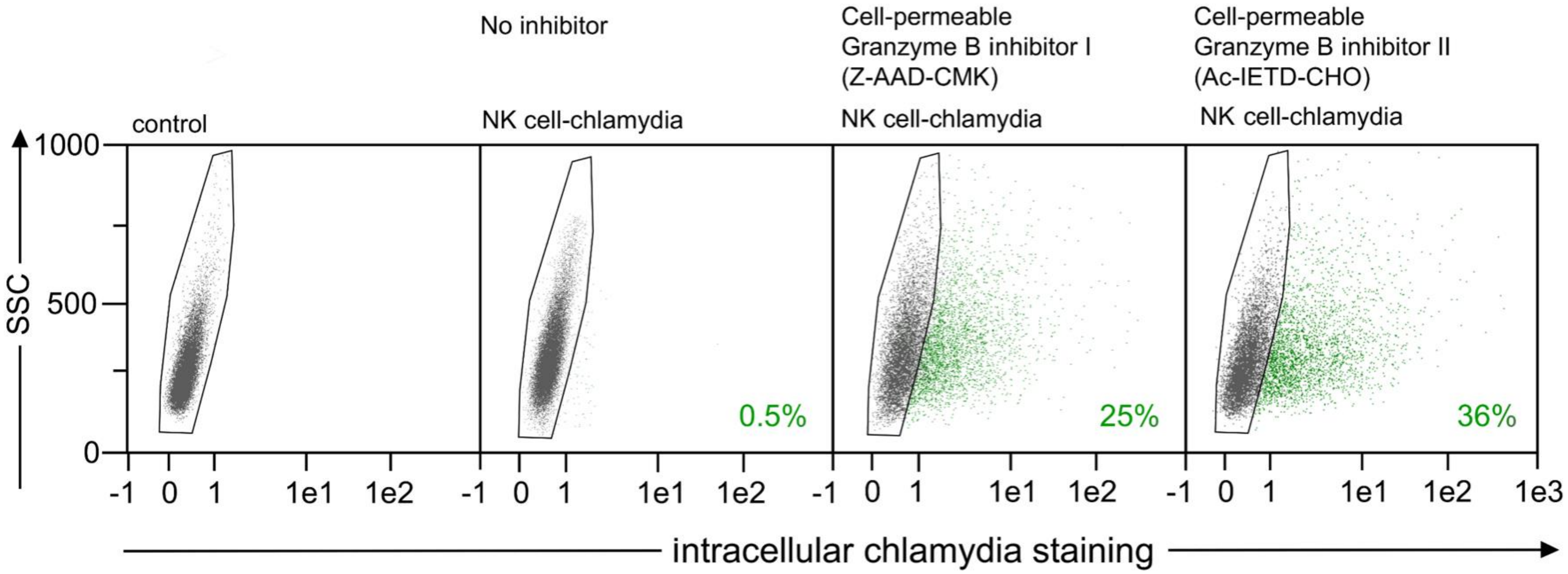

Supplementary Fig. S1. Flow cytometry assay of the infectivity of culture supernatants from chlamydia-infected NK cells. KY-2 cells were treated or not with cell permeable granzyme B inhibitors (100 µM Z-AAD-CMK or 100 µM Ac-IETD-CHO) and infected (MOI 40) for 72 h. Released chlamydia in culture supernatants from infected and non-infected KY-2 cells were harvested by centrifugation and then incubated with BGM reporter cells (48 h), which were finally analysed by flow cytometry. The pellet fraction from the centrifuged culture supernatant of non-infected KY-2 cells was used as a control. To detect/quantify chlamydia-positive cells (green), the negative cell population (black) was identified and gated via corresponding non-infected controls and then subtracted from the total cell population.

Fig. S2

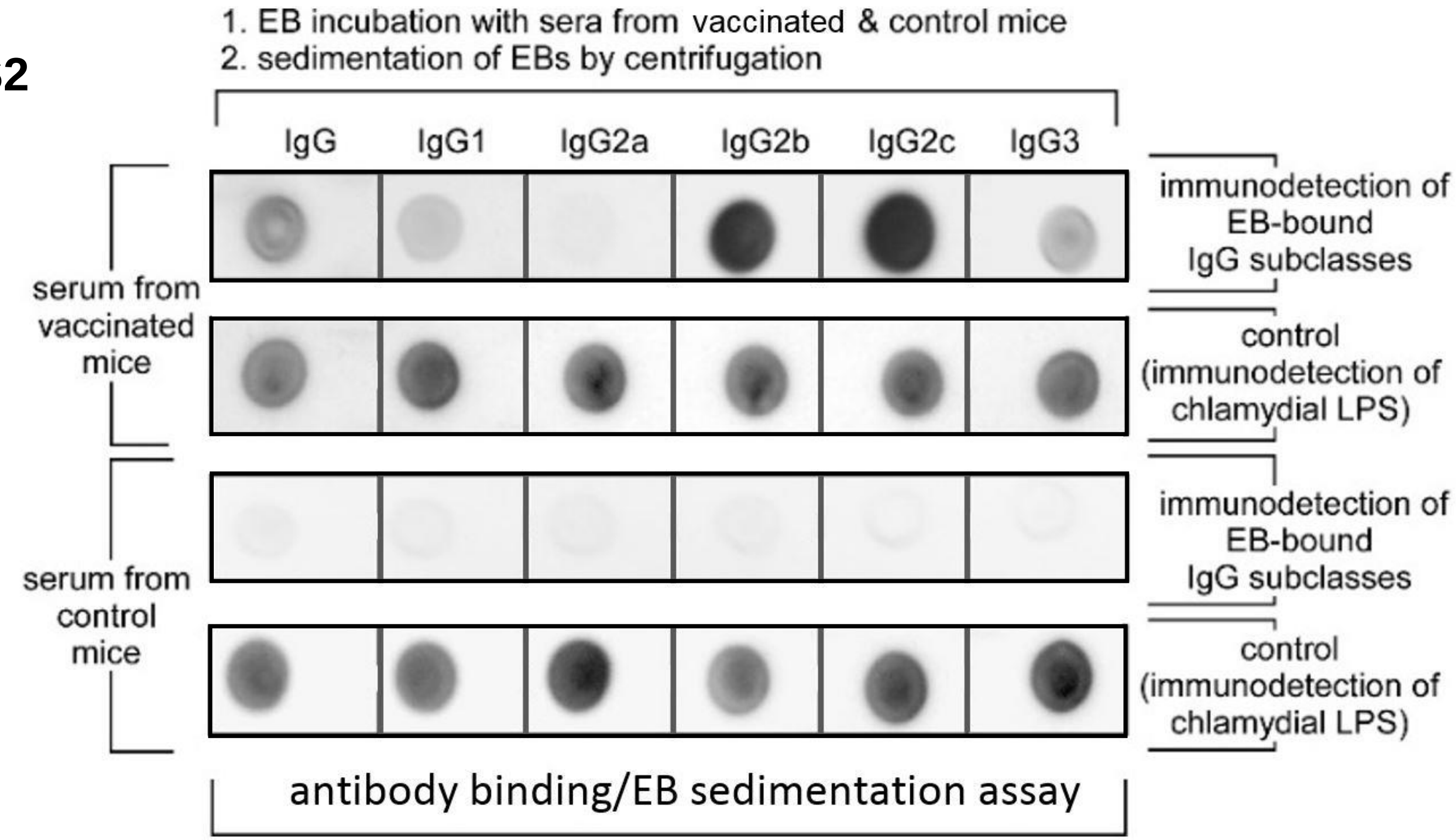

Supplementary Fig. S2. Antibody recognition of isolated/enriched chlamydial EBs. IgG binding to EBs was controlled by an antibody binding/EB sedimentation assay, in which serum-pre-incubated EBs (4 h) were sedimented by centrifugation (30.000xg, 60 min) and analysed in immune-dot-blots probed for IgG subclasses and chlamydial LPS. The figure depicts a cropped blot obtained by each protein evaluation. The full-length blot is shown in the Supplement Figure S18.

Fig. S3

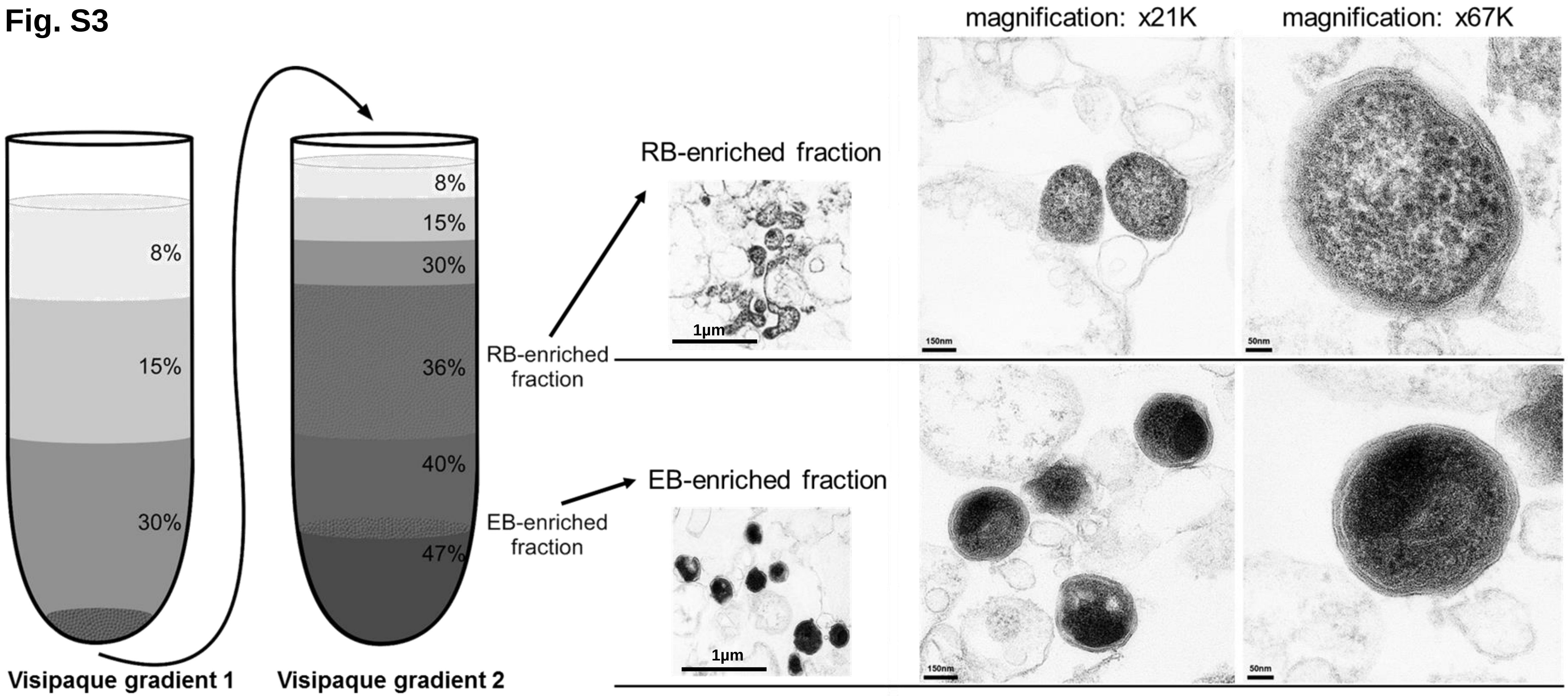

Supplementary Fig. S3. EB- and RB-enrichment fractions checked by TEM. After 48 h cultivation, chlamydia-infected epithelial cells (BGM) were harvested and sonicated in an ultrasonic bath. Chlamydial EBs and RBs in the resulting suspension were purified/enriched by Visipaque gradient centrifugation (left panel) and then resuspended in sucrose-phosphate-glutamic acid buffer (SPGA). The obtained two chlamydial fractions were fixated and embedded in low-melting-point agarose. After preparation and postfixation of small pieces, ultrathin sections were stained and then analysed with a Tecnai-Spirit TEM (FEI) (see methods). Fig. S3 (right panel with different magnifications and/or areas) shows representative TEM images of the analysed bacterial samples. In addition to isolated EBs and RBs co-isolated membranes were visible as bright/pale structures.

Fig. S4

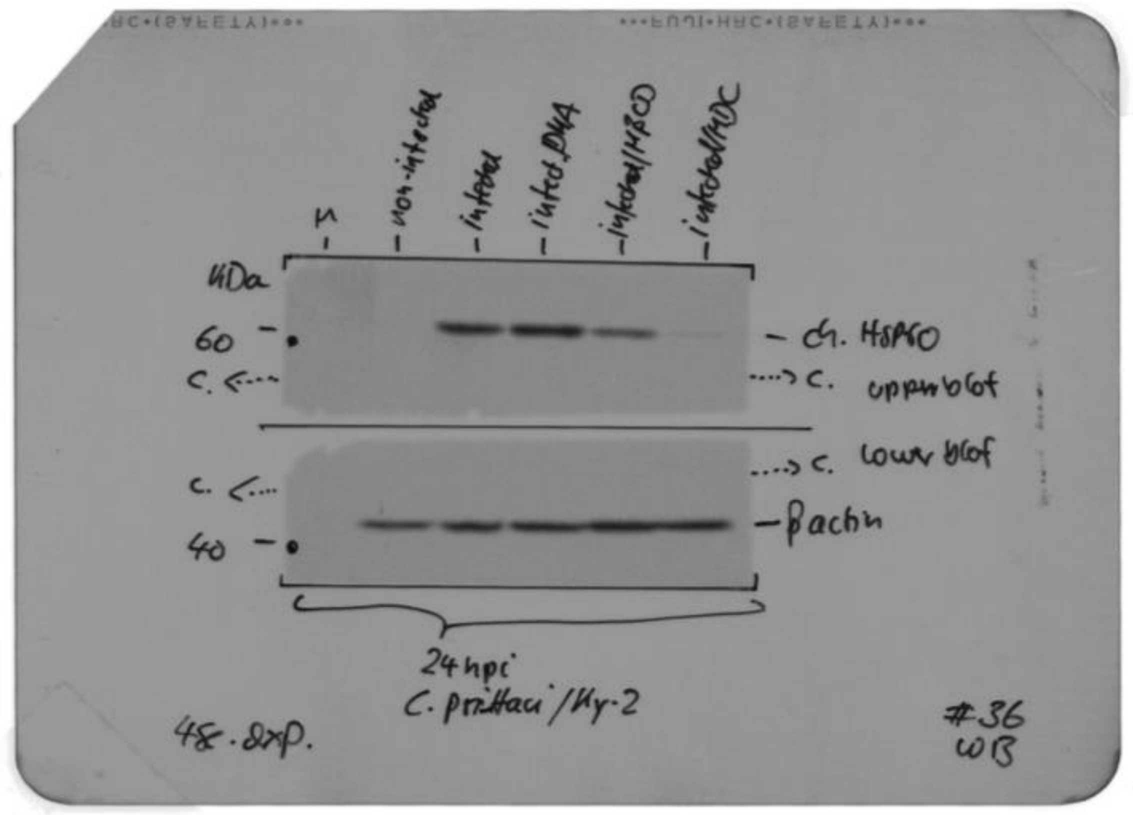

Supplementary Fig. S4. Blot immunostained with antibodies against chlamydial HSP60 (chlHSP60) (upper part) and β-actin (lower part). Lysates (24 hpi) corresponding to 1x10^5 KY-2 cells were loaded onto the SDS-gel. After the run, the gel/blot was horizontally cut into two parts. The samples were from five experiments (non-infected, infected, infected/DMA, infected/MβCD and infected/MDC) and the two blots of chlHSP60 and β-actin (upper & lower part) were developed under the same experimental conditions. (M: Recombinant SDS PAGE Protein Marker, Serva).

Fig. S5

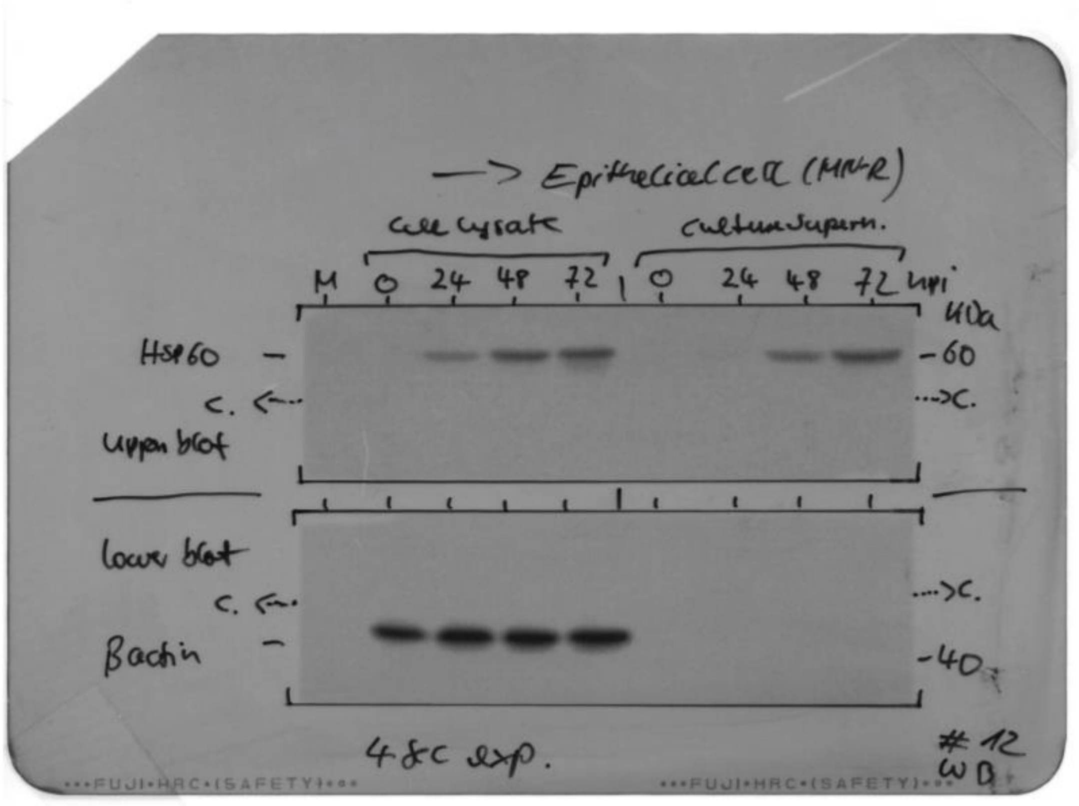

Supplementary Fig. S5. Blots immunostained with antibodies against chlHSP60 (upper part) and β-actin (lower part). Lysates (0-72 hpi) corresponding to $1x10^5$ epithelial MN-R cells and respective cell equivalent amounts of centrifuged culture supernatants (0-72 hpi) were loaded onto the SDS-gel. After the run, the gel/blot was horizontally cut into two parts. Analysed samples were from the same infection experiment and the two blots (upper and lower part) were developed under the same experimental conditions (M: Recombinant SDS PAGE Protein Marker, Serva).

Fig. S6

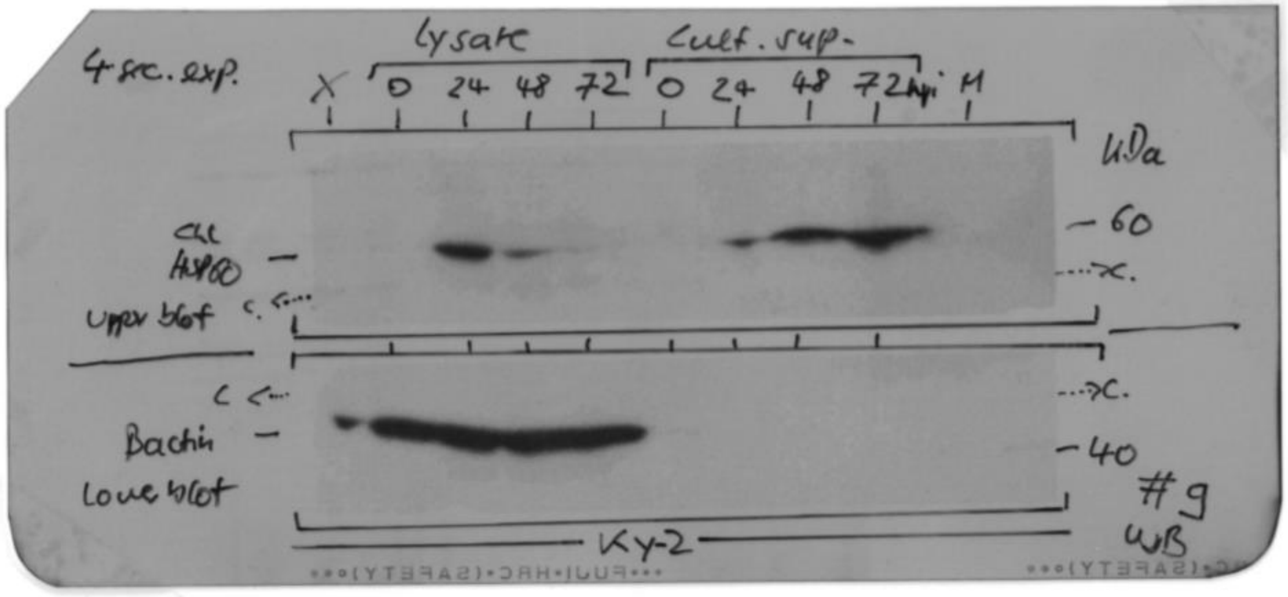

Supplementary Fig. S6. Blots immunostained with antibodies against chlHSP60 (upper part) and β-actin (lower part). Lysates (0-72 hpi) corresponding to $1x10^5$ KY-2 cells and respective cell equivalent amounts of centrifuged culture supernatants (0-72 hpi) were loaded onto the SDS-gel. After the run, the gel/blot was horizontally cut into two parts. Analysed samples were from the same infection experiment and the two blots (upper and lower part) were developed under the same experimental conditions (M: Recombinant SDS PAGE Protein Marker, Serva).

Fig. S7

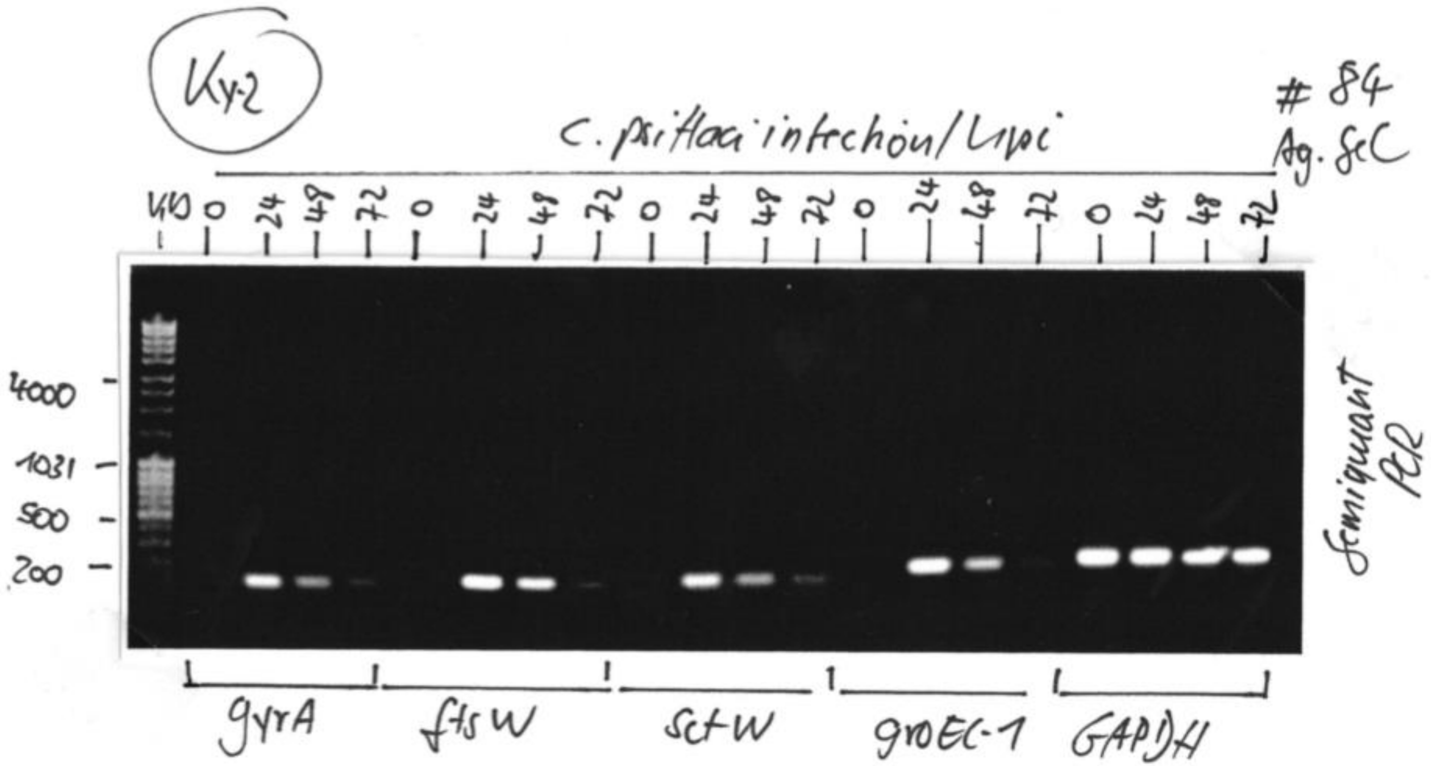

Supplementary Fig. S7. Agarose gel image showing the PCR products of chlamydia infected KY-2 cells (total RNA, 0-72 hpi) amplified using primer sets for gyrA, ftsW, sctN, groEL-1 and GAPDH (Kb: MassRuler DNA Ladder, ThermoFisher Scientific).

Fig. S8

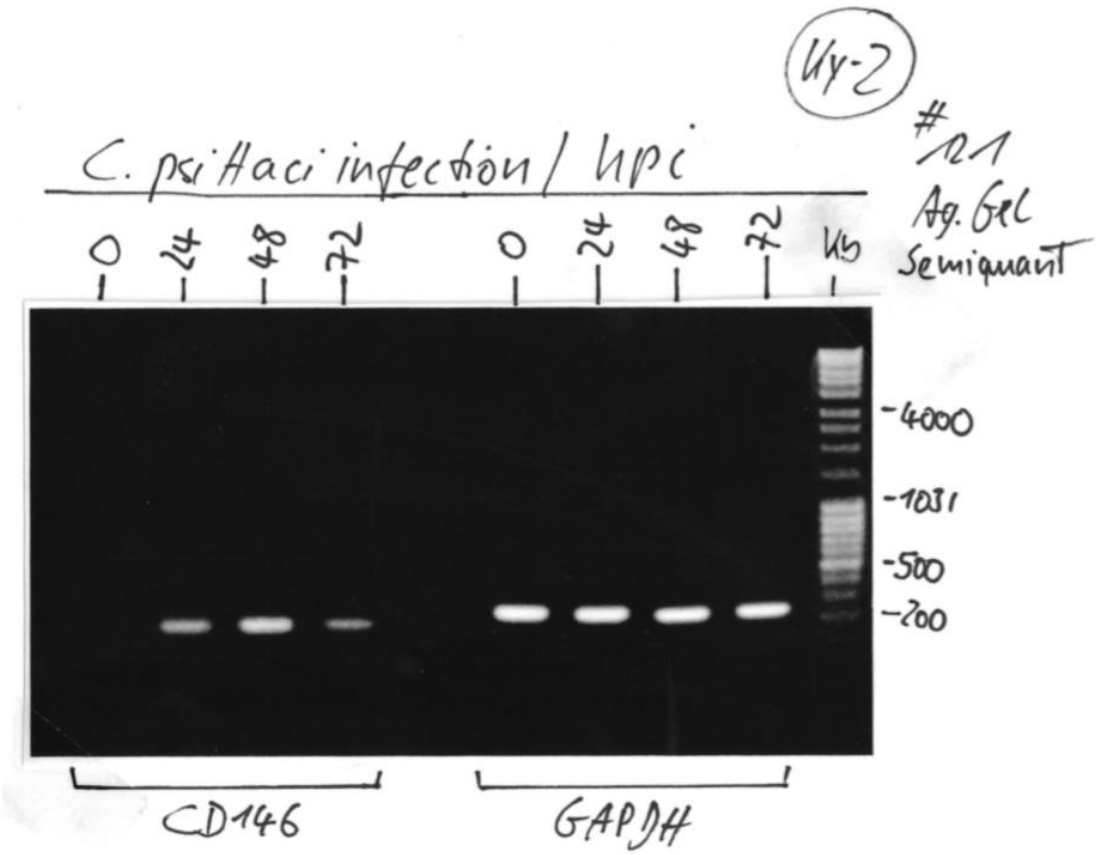

Supplementary Fig. S8. Agarose gel image showing the PCR products of chlamydia infected KY-2 cells (total RNA, 0-72 hpi) amplified using primer sets for CD146 and GAPDH (Kb: MassRuler DNA Ladder, ThermoFisher Scientific).

Fig. S9

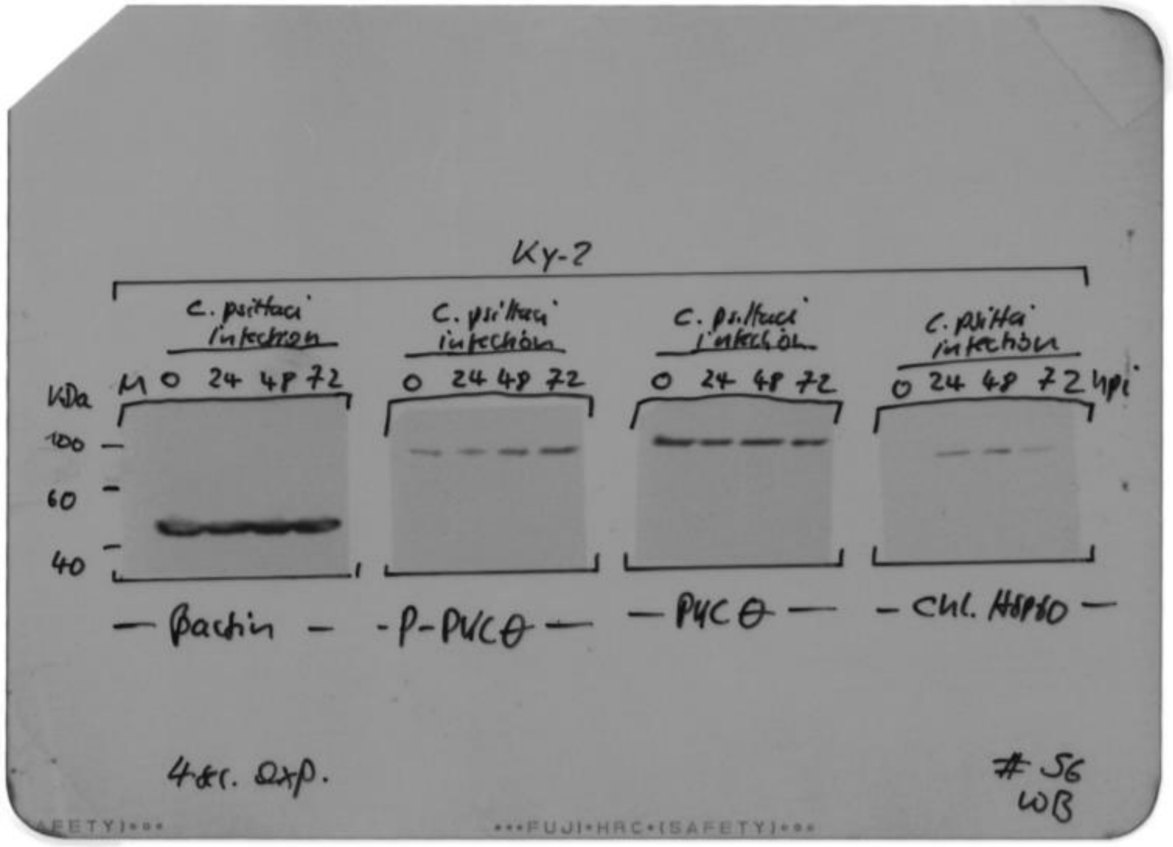

Supplementary Fig. S9. Blots immunostained with antibodies against β-actin, PKCθ, P-PKCθ, and chlHSP60. Lysates (0-72 hpi) corresponding to $1x10^5$ KY-2 cells were loaded onto the SDS-gel. After the run, the gel/blot was vertically cut into four parts. Analysed samples were from the same infection experiment and all four blots were developed under the same experimental conditions (M: Recombinant SDS PAGE Protein Marker, Serva).

Fig. S10

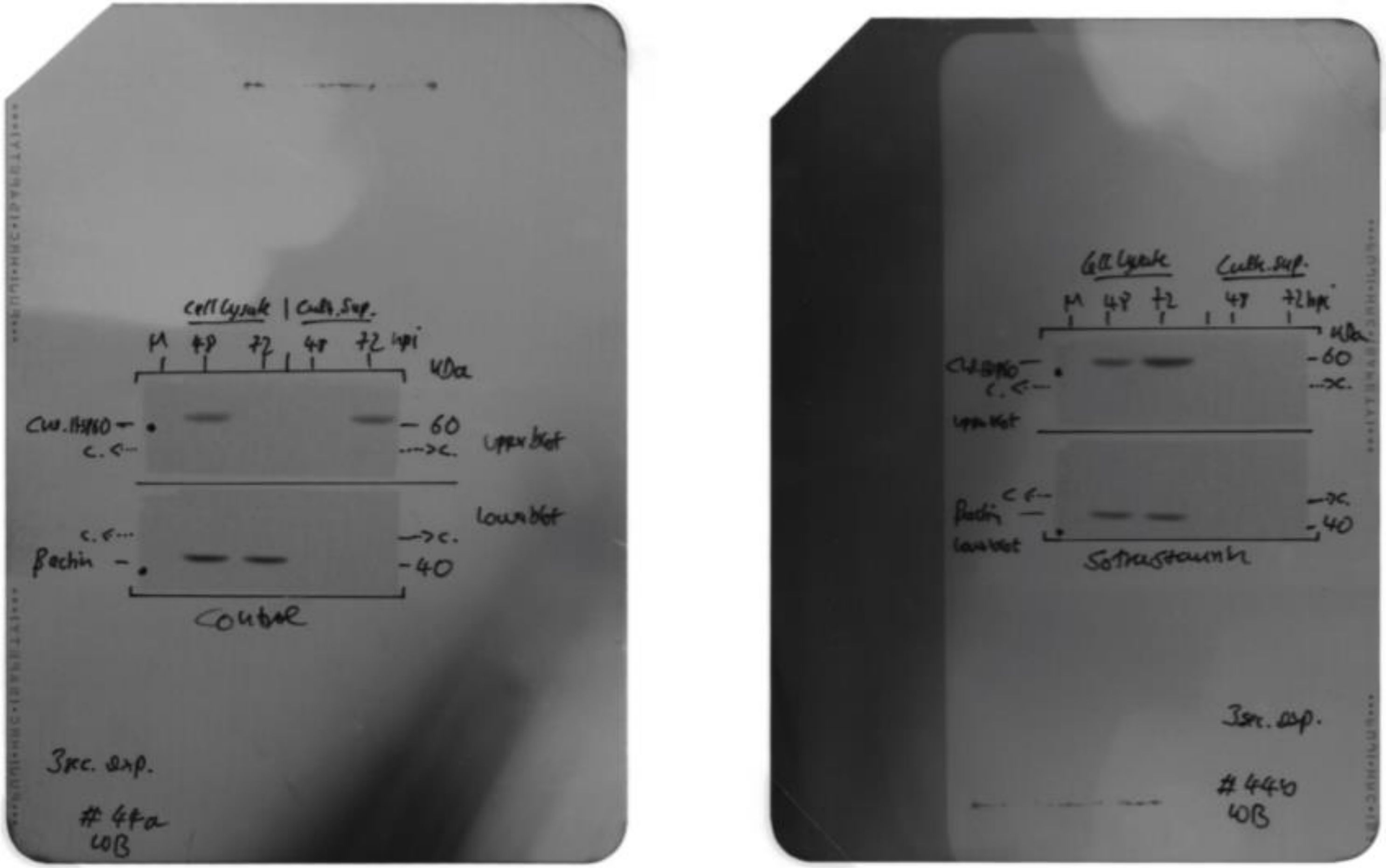

Supplementary Fig. S10. Blots immunostained with antibodies against chlHSP60 and β-actin. Lysates (48 & 72 hpi) corresponding to 1x10^5 KY-2 cells and respective cell equivalent amounts of centrifuged culture supernatants (48 & 72 hpi) were loaded onto the SDS-gels. After the run, the gels/blots were horizontally cut into two parts. Analysed samples were from two infection experiments (left: control & right: sotrastaurin-treatment) and the two blots of chlHSP60 and β-actin (upper and lower part) of each experiment were developed under the same experimental conditions (M: Recombinant SDS PAGE Protein Marker, Serva).

Fig. S11

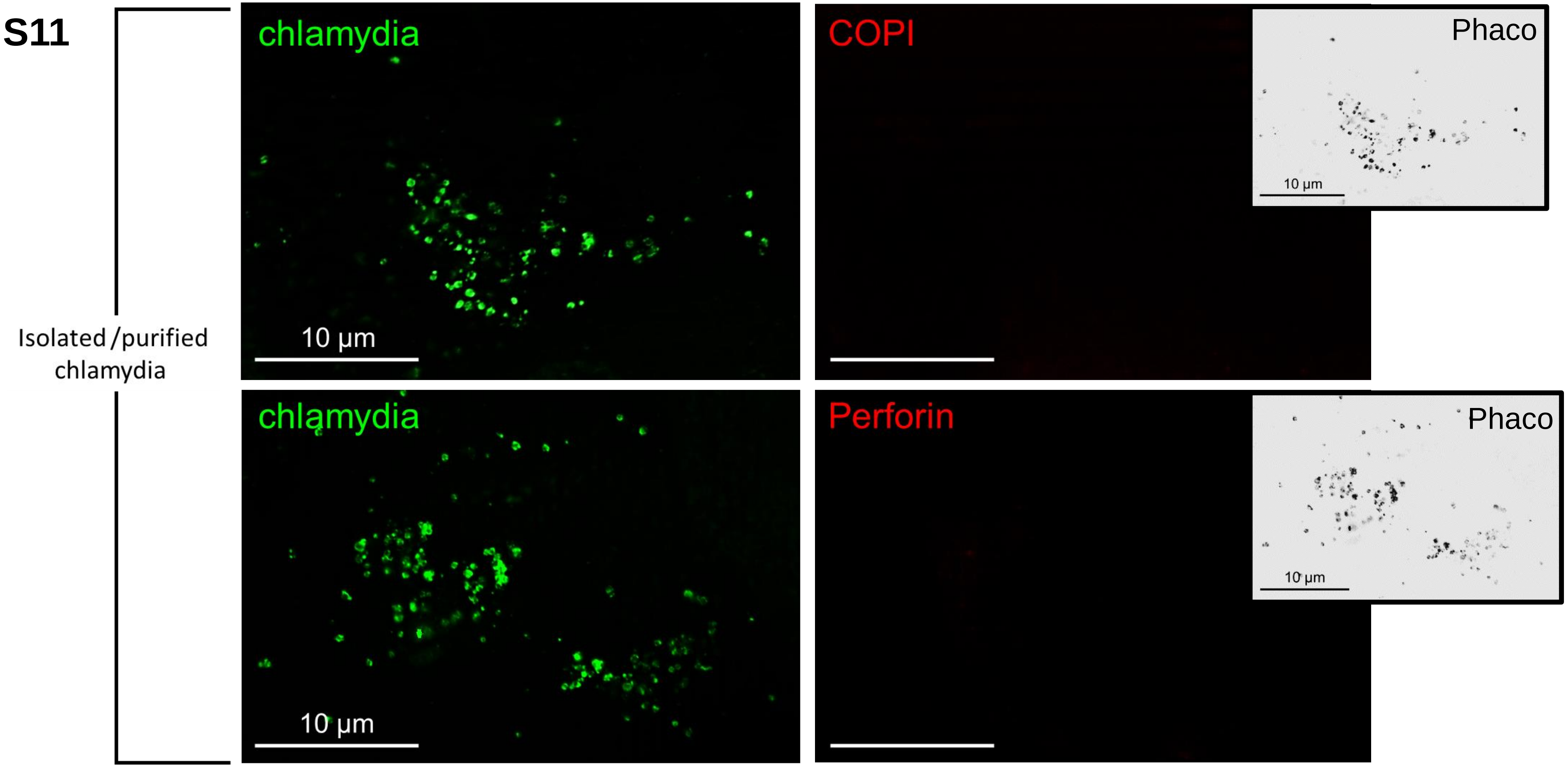

Supplementary Fig. S11. Cross-reactivity of anti-α-COP and anti-perforin antibodies. Control experiments with non-infected KY-2 cells in Fig. 4b show that the anti-chlamydia antibody does not cross-react with COPI and/or perforin-positive cell structures. Here, we additionally checked the possible cross-reactivity of the anti-α-COP and anti-perforin antibodies with isolated/purified *Chlamydia psittaci* from infected epithelial cells (MN-R cells) (first pellet fraction from 8-15-30% Visipaque gradient purification, see Methods). For each analysis, the bacterial fraction (5 µl) was mounted on a microscope slide, carefully dried and fixated with 2% paraformaldehyde. Afterwards, the bacteria were extensively washed with PBS/10 mM glycine and stained with anti-chlamydia (green) as well as anti-perforin (Perforin, red) or anti-α-COP (COPI, red) antibodies. Finally, the immunostained bacterial samples were covered with mounting medium (ThermoFisher Scientific). Images were taken with an Axiovert 200M/ApoTome microscope.

Fig. S12

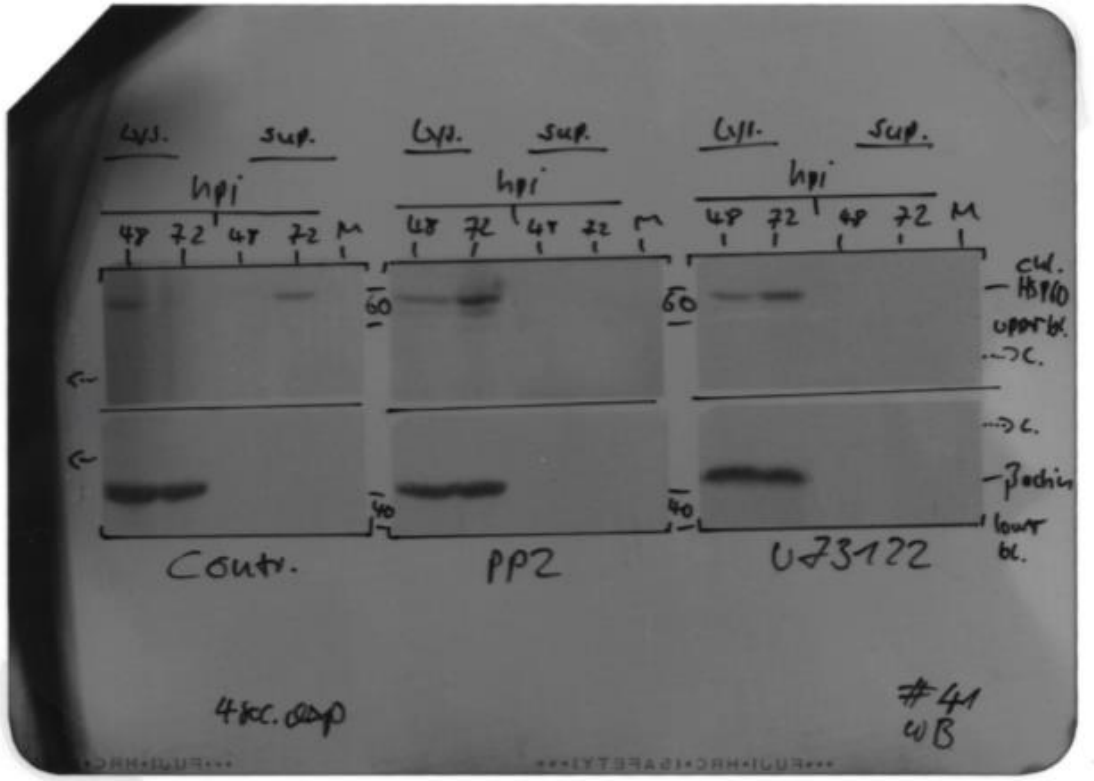

Supplementary Fig. S12. Blots immunostained with antibodies against chlHSP60 and β-actin. Lysates (48 & 72 hpi) corresponding to $1x10^5$ KY-2 cells and respective cell equivalent amounts of centrifuged culture supernatants (48 & 72 hpi) were loaded onto three SDS-gels. After the run, the gels/blots were horizontally cut into two parts. Analysed samples were from three infection experiments (control, PP2- and U73122-treatment) and the two blots of chlHSP60 and β-actin (upper and lower part) of each experiment were developed under the same experimental conditions (M: Recombinant SDS PAGE Protein Marker, Serva).

Fig. S13

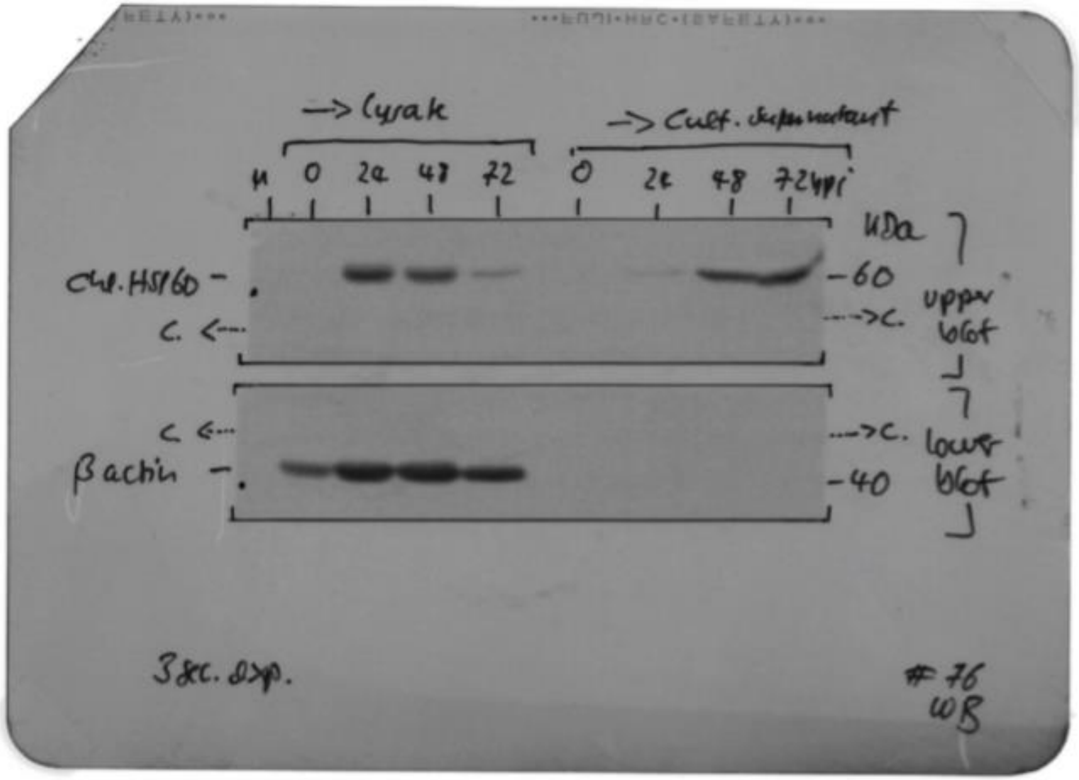

Supplementary Fig. S13. Blots immunostained with antibodies against chlHSP60 and β-actin. Lysates (0-72 hpi) corresponding to $1x10^5$ primary NK cells and respective cell equivalent amounts of centrifuged culture supernatants (0-72 hpi) were loaded onto the SDS-gel. After the run, the gel/blot was horizontally cut into two parts. Analysed samples were from the same infection experiment and the two blots of chlHSP60 and β-actin (upper and lower part) were developed under the same experimental conditions.

Fig. S14

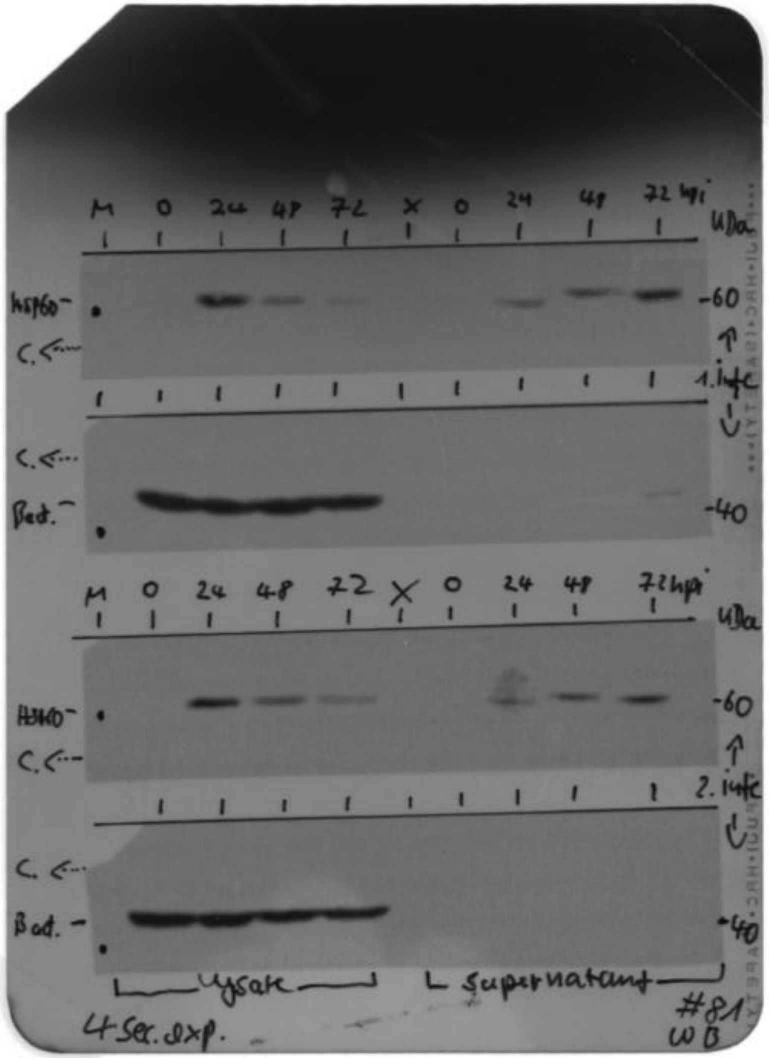

Supplementary Fig. S14. Blots immunostained with antibodies against chlHSP60 and β-actin. Lysates (0-72 hpi) corresponding to 1x10^5 KY-2 cells (1. & 2. chlamydial infection) and respective cell equivalent amounts of centrifuged culture supernatants (0-72 hpi) were loaded onto two SDS-gels (1. & 2. infection). After the run, the gels/blots were horizontally cut into two parts. Analysed samples were from two infection experiments (1. and 2. infection) and the two blots (upper part: chlHSP60 and lower part: β-actin) of each experiment were run under the same experimental conditions. M: Recombinant SDS PAGE Protein Marker, Serva).

Fig. S15

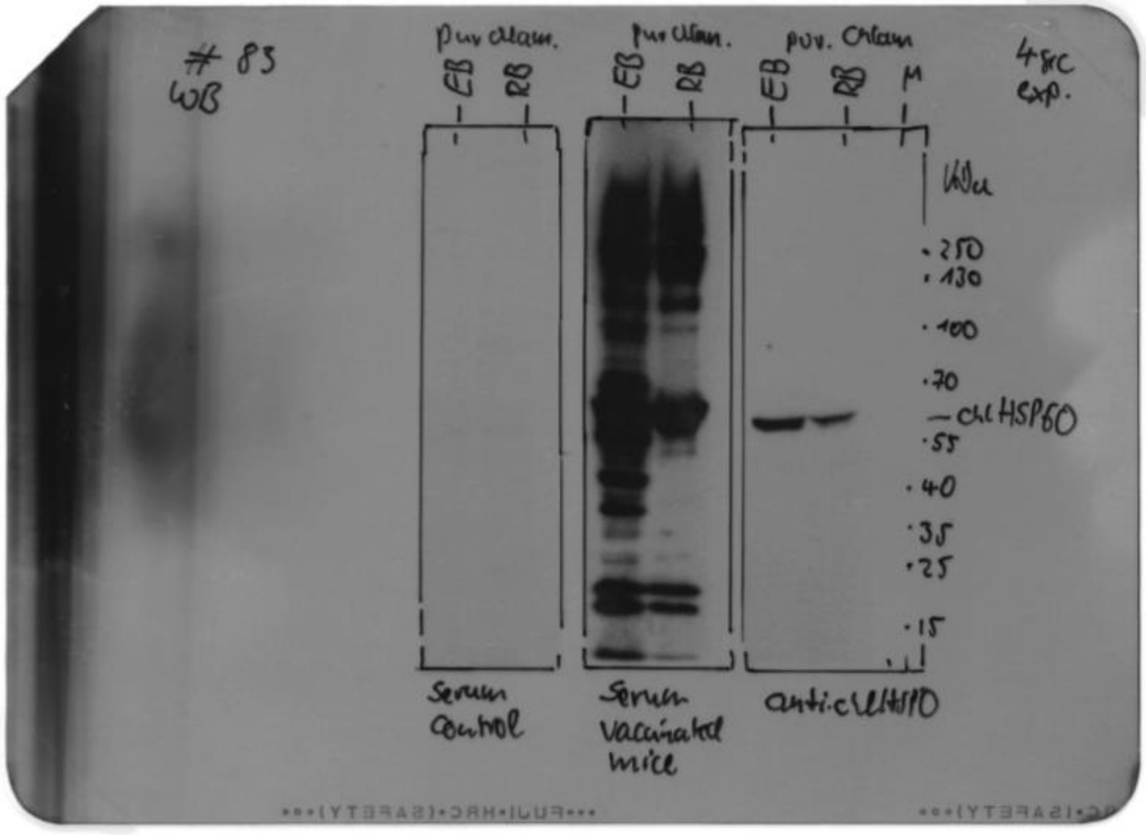

Supplementary Fig. S15. Blots immunostained with control serum (left), vaccination serum (middle) or with antibody against chlHSP60 (right). Purified chlamydial EBs and RBs were loaded onto the SDS-gel. After the run, the gel/blot was vertically cut into three parts. The three blots (control serum, vaccination serum & anti-chlHSP60) were developed under the same experimental conditions (M: PageRuler, ThermoFisher Scientific).

Fig. S16

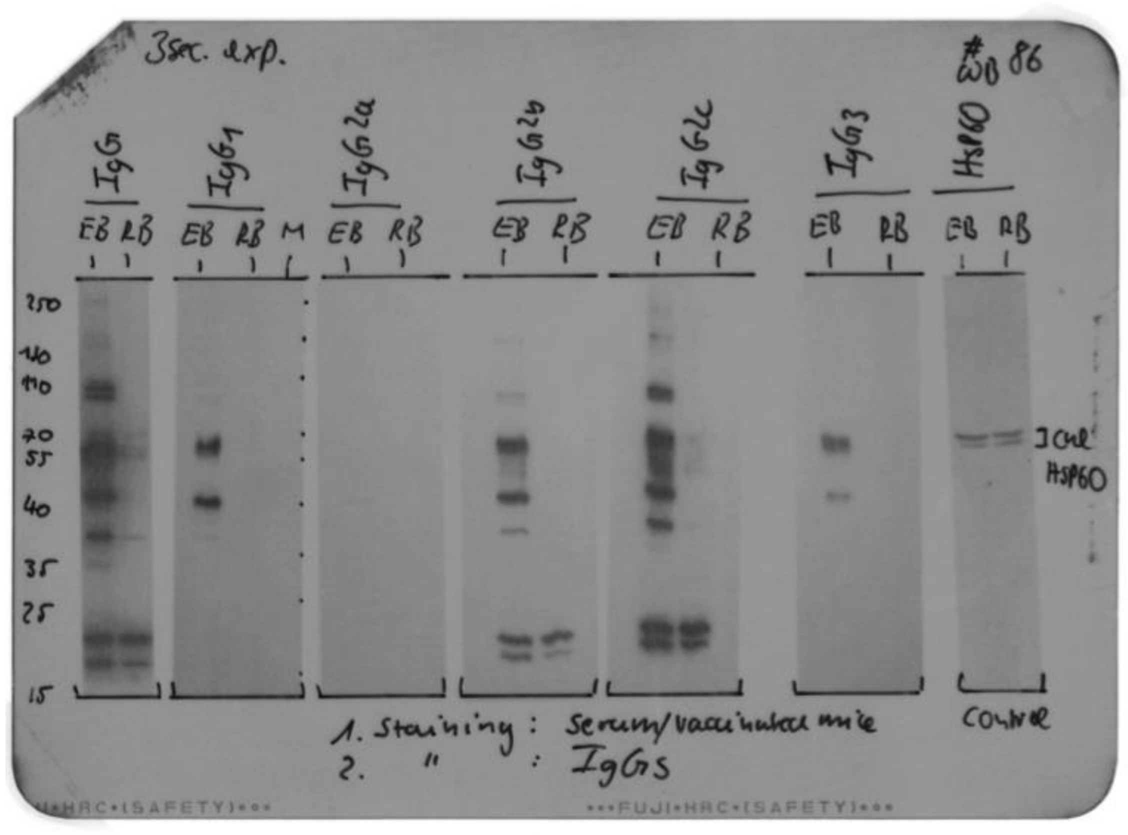

Supplementary Fig. S16. Blots immunostained with vaccination serum or antibody against chlHSP60. Anti-mouse pan-IgG and antibodies specific for the different IgG subclasses (G1, G2a, G2b, G2c and G3) were used as secondary reagents. Purified chlamydial EBs and RBs were loaded onto two SDS-gels (IgG/chlHSP60 and IgG1/G2a/G2b/G2c/G3). After the parallel run, the gels/blots were vertically cut. The seven blots were developed under the same experimental conditions (M: PageRuler, ThermoFisher Scientific).

Fig. S17

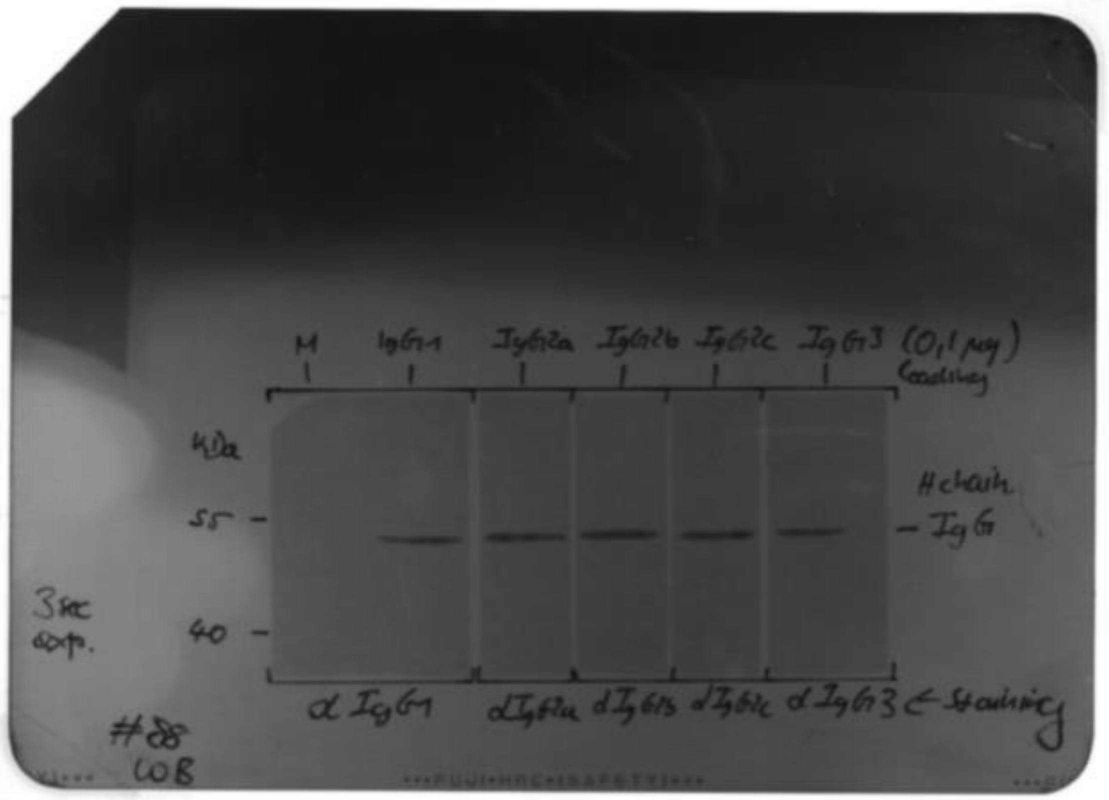

Supplementary Fig. S17. Blots immunostained with antibodies specific for the different IgG subclasses (IgG1, G2a, G2b, G2c and G3). Purified murine IgG subclass proteins were loaded onto the SDS-gel. After the run, the gel/blot was vertically cut into five parts. The five blot parts were developed under the same experimental conditions (M: PageRuler, Thermo Scientific).

Fig. S18

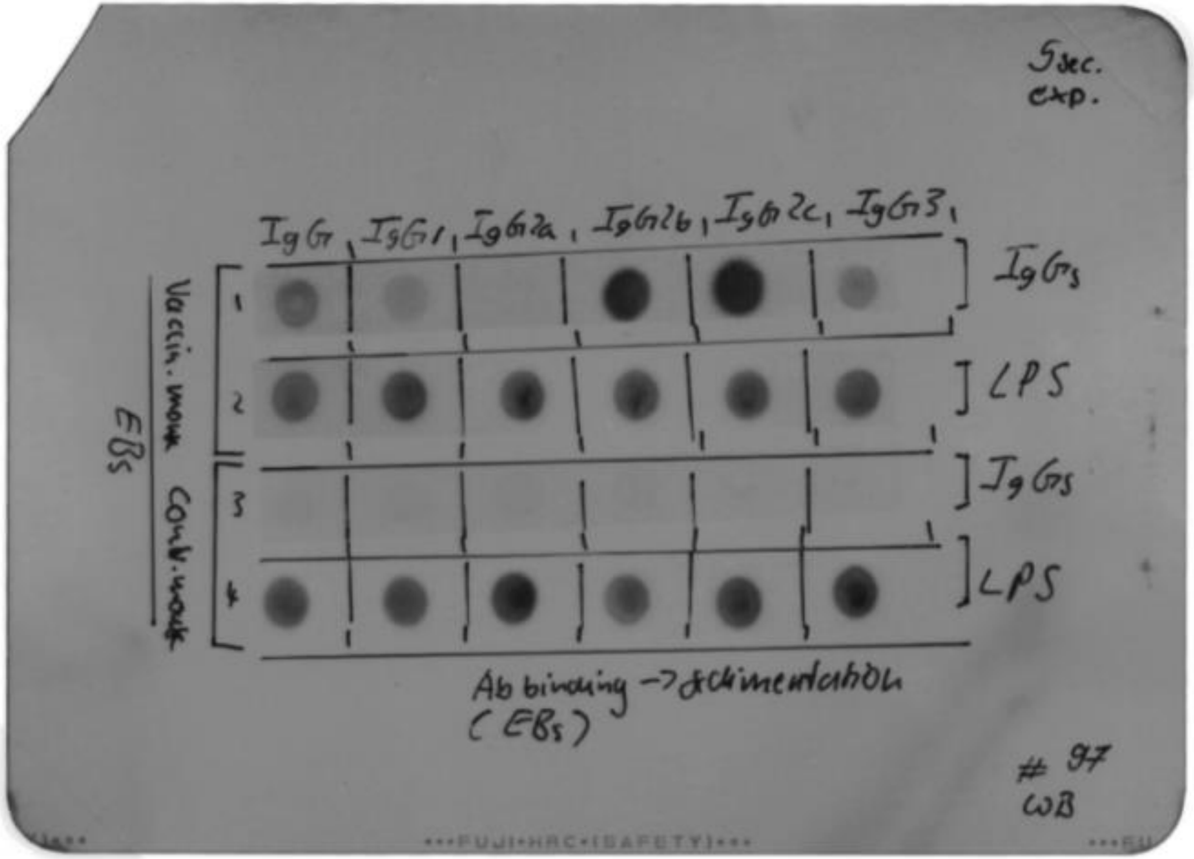

Supplementary Fig. S18. Immuno-dot-blots of the antibody binding/EB sedimentation assay. Vaccination serum-pre-incubated EBs were sedimented by ultracentrifugation and analysed in 24 dot-blots probed for IgG, the five different murine IgG subclasses (IgG1, G2a, G2b, G2c and G3) and chlamydial LPS.
